# Pruned-ADAPT-VQE: Compacting Molecular Ansätze by Removing Irrelevant Operators

**DOI:** 10.1021/acs.jctc.5c00535

**Published:** 2025-09-06

**Authors:** Nonia Vaquero-Sabater, Abel Carreras, David Casanova

**Affiliations:** † 226245 Donostia International Physics Center (DIPC), 20018 Donostia, Euskadi, Spain; ‡ Polimero eta Material Aurreratuak: Fisika, Kimika eta Teknologia Saila, Kimika Fakultatea, Euskal Herriko Unibertsitatea (EHU), PK 1072, 20080 Donostia, Euskadi, Spain; § IKERBASQUE, Basque Foundation for Science, 48009 Bilbao, Euskadi, Spain

## Abstract

The adaptive derivative-assembled problem-tailored variational quantum eigensolver (ADAPT-VQE) is one of the most widely used algorithms for electronic structure calculations in quantum computers. It adaptively selects operators based on their gradient, constructing ansätze that continuously evolve to match the energy landscape, helping avoid local traps and barren plateaus. However, this flexibility in reoptimization can lead to the inclusion of redundant or inefficient operators that have almost zero parameter value, barely contributing to the ansatz. We identify three phenomena responsible for the appearance of these operators: poor operator selection, operator reordering, and fading operators. In this work, we propose an automated, cost-free refinement method that removes unnecessary operators from the ansatz without disrupting convergence. Our approach evaluates each operator after ADAPT-VQE optimization by using a function that considers both its parameter value and position in the ansatz, striking a balance between eliminating low-coefficient operators while preserving the natural reduction of coefficients as the ansatz grows. Additionally, a dynamic threshold based on the parameters of recent operators enables efficient convergence. We apply this method to several molecular systems and find that it reduces ansatz size and accelerates convergence, particularly in cases with flat energy landscapes. The refinement process incurs, at most, a small additional computational cost and consistently improves or maintains ADAPT-VQE performance.

## Graphical abstract

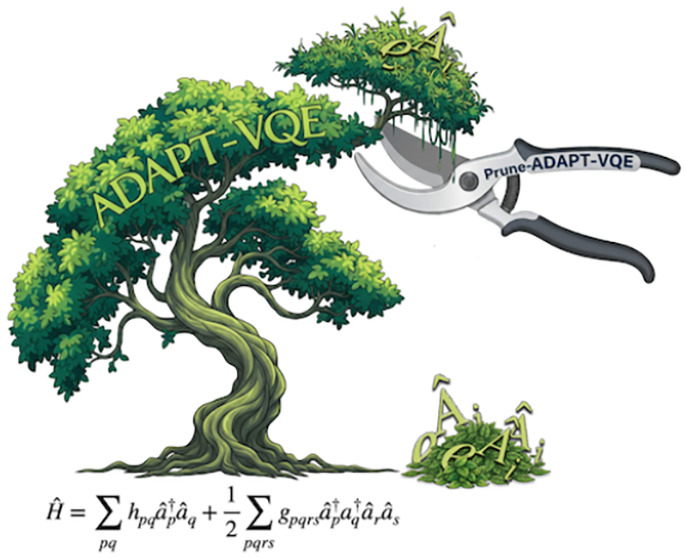

## 1 Introduction

Quantum simulation has long been regarded as one of the most promising applications of quantum computing, with the potential to achieve a significant quantum advantage.^1,2^ One of the earliest quantum algorithms designed for applications in quantum chemistry problems is the quantum phase estimation algorithm (QPEA).^3^ However, QPEA requires deep quantum circuits and extensive use of controlled operations, making it impractical for near-term quantum devices. As an alternative, the Variational Quantum Eigensolver (VQE)^4,5^ was introduced, better suited for the Noisy Intermediate-Scale Quantum (NISQ) era.^6,7^ Despite its advantages, VQE still faces several challenges, including susceptibility to local traps^8,9^ and barren plateaus,^10,11^ which can hinder optimization and convergence.

The effectiveness of VQE strongly depends on the choice of ansatz. An ideal ansatz should be expressive enough to capture the exact solution while remaining shallow enough to be implemented within the coherence time of current quantum hardware. Additionally, it should have a minimal number of parameters to ensure efficient optimization and avoid unnecessary complexity in the classical optimization process. In molecular simulations, chemically inspired ansätze, such as Unitary Coupled-Cluster (UCC) methods,^12,13^ are commonly used. These ansätze encode Fermionic excitations applied to an initial state, such as Hartree–Fock. However, the direct encoding of Fermionic excitations leads to deep circuits with a large number of two-qubit gates, making them impractical for NISQ devices.^14^

To address these limitations, adaptive derivative-assembled problem-tailored VQE (ADAPT-VQE)^15^ was introduced as an iterative and problem-tailored approach. Instead of defining a fixed ansatz, ADAPT-VQE constructs the wave function dynamically by adding one Fermionic excitation per iteration, selecting only the most relevant operators required for convergence. This adaptive strategy significantly reduces circuit depth compared to conventional VQE while ensuring that the ansatz remains compact and efficient. The resulting wave function takes the form

1 $$\left|\Psi \right\rangle =\prod_{i=1}^{N}e^{\theta_{i}{Â}_{i}}\left|\psi_{0}\right\rangle$$

where *N* is the number of selected excitation operators ({*Â*_*i*_}). While standard UCC-based VQE optimizes all parameters {θ_*i*_} simultaneously, ADAPT-VQE builds the ansatz incrementally. At each step, a new parameter is introduced (initially set to zero), while the previously optimized parameters are recycled (their values taken from the previous iteration). Then, all parameters are reoptimized. This strategy has shown to be very efficient, allowing ADAPT-VQE to improve convergence and reduce the likelihood of getting trapped in local minima.^16^ This iterative refinement provides a warm start for parametrized circuits, a strategy demonstrated to be key in enhancing the efficiency of variational algorithms.^17^

Despite its advantages, ADAPT-VQE faces significant challenges on current quantum hardware. As system complexity increases, the corresponding circuit depth grows, eventually exceeding the practical limits of today’s devices.^18^ Also, high noise levels from gate errors, readout inaccuracies, and crosstalk degrade the accuracy of the results.^19^ Moreover, mitigation methods add computational overhead, further limiting ourselves to the simplest techniques.^20^ Thus, while ADAPT-VQE remains a powerful algorithm, its practical application to significant molecular systems is currently infeasible due to circuit depth, amount of measurements, noise, and hardware constraints, requiring significant advancements in quantum technology.^21^ Further algorithmic improvements aimed at reducing circuit depth are essential to enable its implementation on near-term quantum devices.^22–25^

The ADAPT-VQE method employs a gradient-based criterion to select the next excitation operator at each iteration, adding to the ansatz the operator with the largest gradient. This strategy has been shown to mitigate optimization challenges associated with barren plateaus.^16^ However, the gradient-based criterion is not infallible. The operator with the highest gradient does not always lead to the greatest energy reduction, and in some cases, its contribution to the energy can be negligible, with nearly vanishing parameter values (see Supporting Information, Section S7). Moreover, during the iterative process, some operators that initially had a significant coefficient may eventually become negligible, contributing little to the total energy while increasing the overall ansatz size.

Since such situations cannot be anticipated in advance, the gradient-driven, one-operator-at-a-time ansatz construction remains the most effective strategy currently available in ADAPT-VQE. However, one may wonder if there is some practical scheme that can, at least partially, correct this situation. In this work, we explore a postselection strategy aimed at removing negligible operators in order to reduce the size of the ansatz while preserving most of its expressivity. This idea has been previously explored,^26^ where under-performing operators were removed or blocked during several ADAPT-VQE iterations, leading to mixed results. Here, we propose an alternative protocol based on removing operators according to two simple criteria: The position of the operator within the ansatz and the magnitude of its associated coefficient. This approach is specifically designed with current NISQ devices in mind, where ansatz size has a significant impact on computational accuracy.

This study has two main objectives: (i) to identify and understand scenarios where certain excitation operators appear to perform poorly, exhibiting nearly zero-valued parameters, and (ii) to develop an efficient protocol for eliminating these redundant operators, thereby constructing more compact ansätze that yield shorter quantum circuits without compromising energy accuracy.

## 2 Spotting Superfluous Operators

To begin, we aim to characterize cases where certain excitation operators contribute negligibly to the ansatz energy, exhibiting near-zero associated parameters. To illustrate this issue, we investigate the performance of ADAPT-VQE in a stretched linear H_4_ system (interatomic distance of 3.0 Å). Highly correlated systems like this are particularly challenging, often requiring long ansätze to achieve chemical accuracy. For our simulations, we employ the 3-21G basis set, consisting of 8 orbitals (Figure S1) mapped to 16 qubits. Although ADAPT-VQE simulations in the literature commonly employ the minimal STO-3G basis set, in this study we opt for the larger 3-21G basis. This allows for more complex wave function construction and better recovery of electron correlation. For comparison, representative results using the STO-3G basis can be found in the Supporting Information (Section S10).

The excitation operator pool consists of UCC operators restricted to occupied-to-virtual spin-singlet adapted single and double excitations.^15^ We write these operators as *Â*_*i*_^*a*^ and *Â*_*ij*_^*ab*^ for single and double excitations, respectively, where *i*,*j* represent the occupied orbitals in the Hartree–Fock determinant and *a*,*b* span all virtual orbitals. For simplicity, along the text, we use the Mulliken notation for the orbitals abbreviated as *ng* and *nu*, where *n* = 1–4 is the orbital’s number, and *g* denotes gerade and *u* ungerade symmetry. See Section S1 for additional information. Explicit expressions for the spin-adapted unitary Fermionic operators in terms of creation and annihilation operators can be found in the Supporting Information (Section S1). The Fermionic spin-adapted operators are transformed to qubit operators using the Jordan–Wigner mapping.^27^ It should be emphasized that, under this mapping, a single Fermionic operator generally corresponds to a linear combination of multiple qubit operators. In practice, these qubit operators are implemented in quantum circuits through a Trotterization scheme with a limited number of Trotter steps. However, this approximation can break the spin symmetry of the ansatz, potentially leading to solutions that are strictly not spin-pure. For completion, we have also tested our protocol with other pools^22^ and basis sets. The results can be found in the Supporting Information (Sections S9 and S10).

Numerical optimization of the ansatz parameters is carried out using the Broyden–Fletcher–Goldfarb–Shanno algorithm.^28^ Simulations are conducted with an in-house Python implementation of ADAPT-VQE, openly available on GitHub,^29^ utilizing the NumPy,^30^ SciPy,^31^ and OpenFermion^32^ packages. All simulations in this study were performed under idealized conditions, neglecting both sampling and hardware-induced noise.

We define three convergence criteria for our simulations:

1. The optimized coefficient of the most recently added operator becomes zero.
2. The energy improvement is nonpositive.
3. A zero-valued coefficient is added or the last operator is removed, which effectively returns the ansatz to its previous form, causing the same operator to be selected and subsequently discarded.

The simulation is considered converged when any of these criteria are met for ADAPT-VQE, or when only criteria 1 and 3 are met for Pruned-ADAPT-VQE.

Figure 1a illustrates the evolution of the energy error in ADAPT-VQE relative to full configuration interaction (FCI) as a function of the number of operators in the ansatz. The distribution of the absolute value of the ansatz parameters at *N* = 69 (Figure 1b) reveals the presence of several operators with nearly zero-valued parameters. Notably, contiguous operators with negligible parameter values correspond to flat regions in the energy profile, indicating that, despite being selected based on the gradient criterion, these operators do not meaningfully contribute to energy optimization. Consistently, in Section S11 we provide additional data showing the correlation between the magnitude of a parameter and the contribution of the associated operator to the energy.

**Figure 1:**
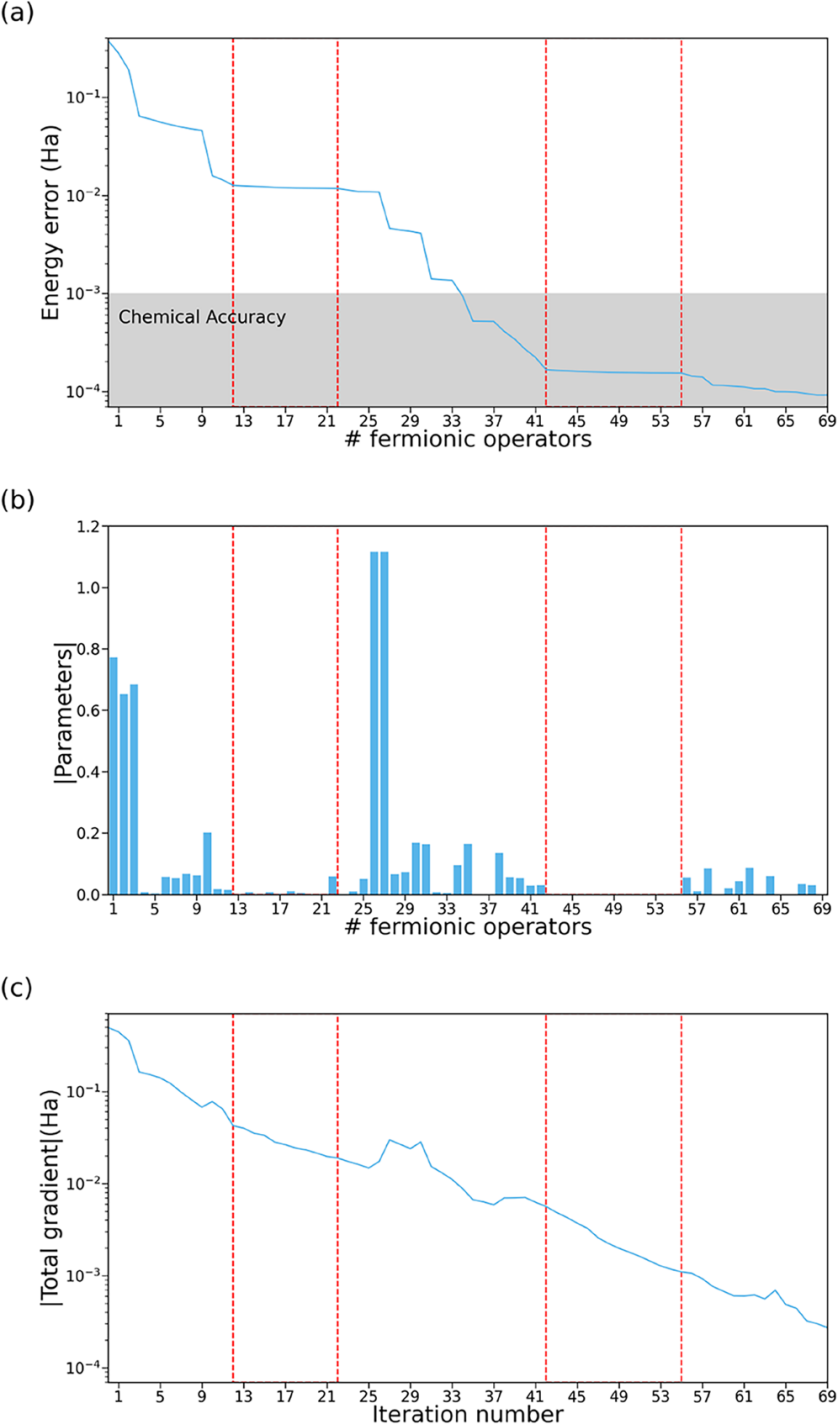
(a) Energy error (in a.u.) of the ADAPT-VQE with respect to FCI as a function of the number of operators, (b) distribution of absolute value of the ansatz parameters at *N* = 69, and (c) the norm of the total gradient (sum of the gradients of all the operators in the pool) (in a.u.) obtained for linear H_4_ with interatomic distance of 3.0 Å, with the 3-21G basis set. Vertical dashed red lines delimit flat energy regions.

Figure 1c shows the gradient norm as a function of the iteration number, revealing the presence of gradient troughs^33^ corresponding to the flat regions in the evolution of energy errors (Figure 1a). As expected, the gradient norm increases concurrently with the parameter values, while the energy error decreases.

In Section S11 we present additional data that demonstrates the correlation between the parameter value and the contribution of the associated operator to the energy.

A detailed analysis of the occurrence of operators with θ ≈ 0 in the ADAPT-VQE ansatz for the linear H_4_ simulation reveals three underlying mechanisms: (i) poor or incorrect operator selection, (ii) operator reordering, and (iii) fading operators. A similar analysis for the water molecule can be found in the Supporting Information (Section S6).

### 2.1 Poor Operator Selection

Typically, after adding a new operator and reoptimizing all the parameters, using the previously optimized values as initial guesses in the classical optimization (a technique we refer to as amplitude recycling), the ansatz gains expressivity, leading to a lower energy and reduced error relative to the exact solution. However, in some cases, the newly added operator *Â*_*N*_ has little to no impact on the ansatz, resulting in a nearly zero parameter value θ_*N*_ ≈ 0 from the moment it is introduced, and failing to significantly alter the energy. We classify these instances as a consequence of poor (or incorrect) operator selection, since more optimal paths can be found (see Supporting Information, Section S7). The selection of these operators appear to be closely related to the presence of gradient troughs, which hinder the search for the optimal operator and result in regions with minimal error improvement, i.e., flat areas.

As illustrated in Figure 2, certain operators exhibit nearly zero parameter values immediately after being introduced into the ansatz, indicating that their selection did not meaningfully contribute to the optimization process.

**Figure 2:**
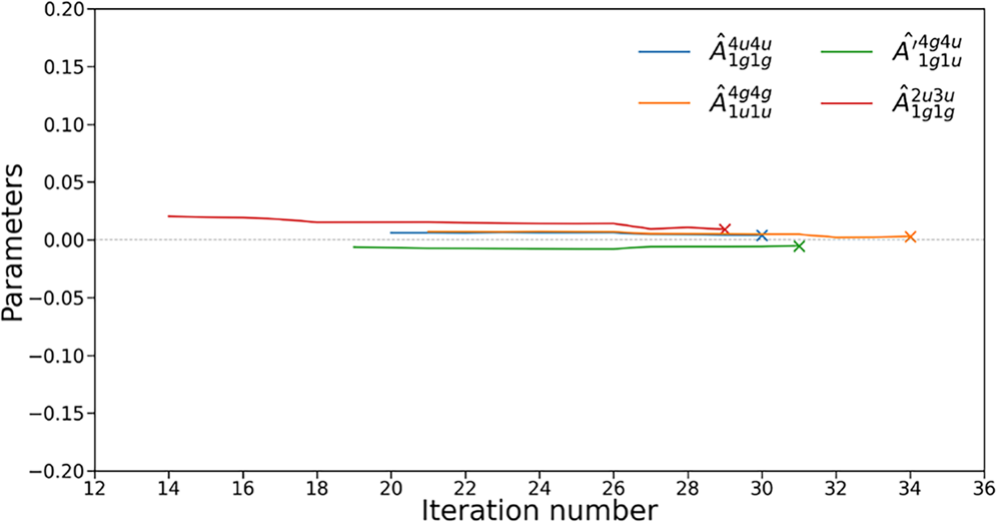
Parameter values of various operators from their introduction to iteration 34 for the simulation of linear H_4_ with interatomic distance of 3.0 Å, with the 3-21G basis set. Cross markers indicate when the operator is removed by Pruned-ADAPT-VQE (see Section 4).

These operators exhibit consistently small parameter values from the moment they are introduced, remaining negligible throughout the entire simulation. This suggests that they were poorly or incorrectly selected and could potentially be removed from the ansatz without significant impact. To verify this, we compare the ADAPT-VQE energy at iteration 27 (just after the energy drop observed in Figure 1) with the energy obtained after eliminating these operators, *Â*_1*g*1*g*_^4*u*4*u*^ and *Â*′_1*g*1*u*_^4*g*4*u*^, and reoptimizing the remaining parameter values. The resulting energy difference is minimal, approximately 0.046 mHa, reinforcing the need to develop strategies for identifying and eliminating inefficient operators.

### 2.2 Operator Reordering

The adaptive nature of ADAPT-VQE allows the ansatz to dynamically adjust to the specific requirements of the problem being studied. As the wave function is iteratively constructed by adding excitation operators, the ansatz continuously evolves to improve accuracy. Notably, once an operator is added to the ansatz, it remains in the pool and is not removed.^15^ This can lead to the inclusion of multiple instances of the same operator. While such duplicity can sometimes be beneficial, we have identified cases where adding a duplicate operator causes a sudden drop in the parameter value of the previously included instance to near-zero value, no longer effectively contributing to the final wave function. This behavior serves as an effective reordering mechanism, highlighting ADAPT-VQE’s ability to dynamically optimize the sequence of operators, an important feature given that excitation operators from UCC generally do not commute, i.e., different orderings of operators are not equivalent.^34^

Figure 3 illustrates this reordering effect, showing the progression of operator *Â*_1*g*_^3*g*^, which has been added four different times. In iteration 23, the second *Â*_1*g*_^3*g*^ is added, causing a sudden drop in the first operator parameter value. This situation is repeated in iteration 37, when a third operator is added, causing the second *Â*_1*g*_^3*g*^ coefficient to drop to almost zero. This shows how operator *Â*_1*g*_^3*g*^ is relocated as the ansatz progresses. This suggests that the ansatz dynamically adjusts the sequence of operators, effectively reorganizing them to better optimize the wave function construction.

**Figure 3:**
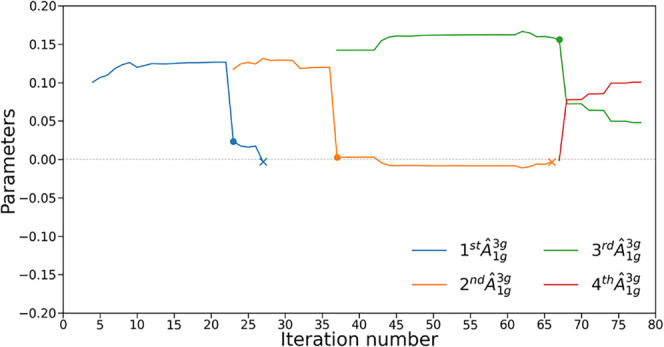
Parameter values of various operators from their introduction to iteration 80 in the simulation of linear H_4_ with interatomic distance of 3.0 Å and the 3-21G basis set. Full circles indicate introduction of another instance of the operator. Cross markers indicate when the operator is removed by Pruned-ADAPT-VQE (see Section 4).

In other cases, however, we observe that adding an additional instance of the same operator does not cause the parameter of the previous instance to drop to zero. Instead, the two instances share similar parameter values, such that their sum remains close to the value of the original parameter. This behavior suggests a form of quasi-commutation between that segment of the ansatz and the repeated operator, where placing the operator in either position results in a similar energy. Consequently, the optimizer converges to an intermediate configuration in which both instances retain a significant portion of the parameter. This phenomenon is observed in Figure 3, where the operator *Â*_1*g*_^3*g*^ is added for a fourth time.

### 2.3 Fading Operators

As the ansatz grows, some operators that initially play a significant role, having sizable parameter values, gradually become irrelevant. In other words, certain operators that were once crucial for describing the wave function eventually contribute little to the final solution. Predicting when such recalibrations will occur is generally challenging. One possible explanation is that the ansatz may initially converge to a local minimum where a given excitation operator is essential. However, as the ansatz expands and explores a larger Hilbert space, the algorithm may transition to a lower-energy minimum where the previously important operator is no longer needed, leading to a near-zero parameter value. Additionally, this fading effect could be linked to the “burrowing into the energy landscape” process,^16^ where qualitative changes in the wave function structure might render certain operators obsolete. Figure 4 exemplifies this phenomenon, showing how operators *Â*_1*g*1*u*_^2*g*4*u*^ and *Â*_1*g*1*u*_^4*g*2*u*^, introduced in iterations 10 and 11 of ADAPT-VQE, initially carry significant weight but become negligible after iteration 30.

**Figure 4:**
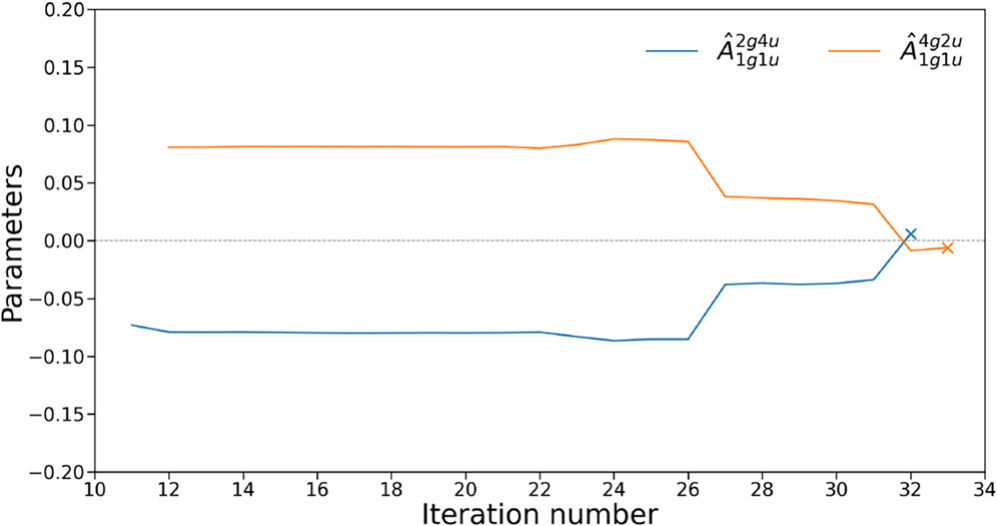
Parameter values of various operators from their introduction to iteration 34 in the simulation of linear H_4_ with interatomic distance of 3.0 Å, with the 3-21G basis set. Cross markers indicate when the operator is removed by Pruned-ADAPT-VQE (see Section 4).

### 2.4 Cooperative Operator Action

We observe that, in some cases, newly added operators initially have negligible parameter values but later become significant contributors to the ansatz. While their inclusion may initially appear to be a poor selection, the addition of subsequent operators can trigger a substantial increase in their parameter values. This suggests that certain operators, despite their seemingly insignificant impact at first, play a crucial role in unlocking specific regions of the Hilbert space. Their effectiveness emerges only when combined with other operators, highlighting the necessity of collective and cooperative action in the construction of an optimal ansatz.

This phenomenon is clearly illustrated in Figure 5, which depicts the ansatz composition at iterations 26 and 27. These iterations coincide with a sudden energy drop (Figure 1a), demonstrating how the cooperative action of multiple operators can unlock lower-energy solutions that were previously inaccessible.

**Figure 5:**
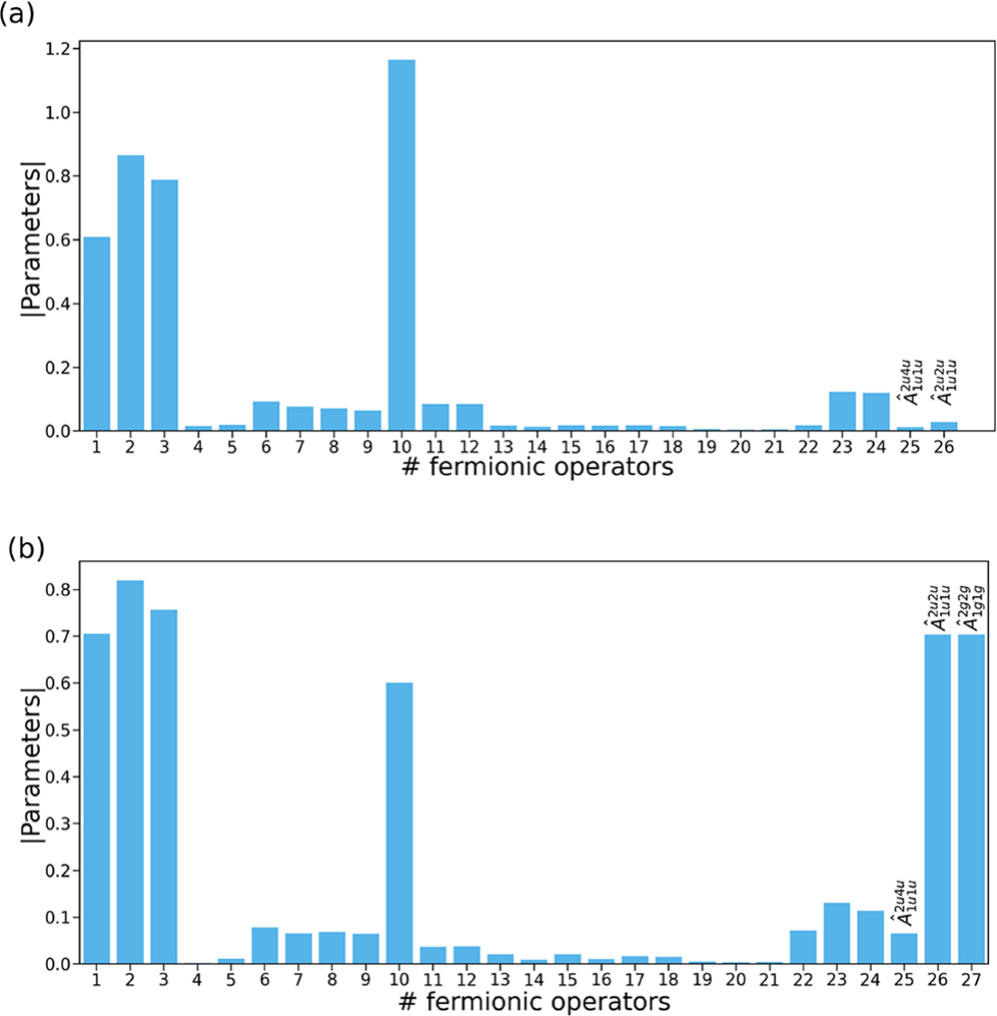
Absolute parameter value at iteration 26 (a) and 27 (b) of ADAPT-VQE for the simulation of linear H_4_ with interatomic distance of 3.0 Å, with the 3-21G basis set.

Operator *Â*_1*u*1*u*_^2*u*2*u*^ in position 26 (and, to a lesser extent, operator *Â*_1*u*1*u*_^2*u*4*u*^ in position 25) initially appears with a small parameter value. However, after an additional ADAPT iteration, specifically, upon the introduction of operator *Â*_1*g*1*g*_^2*g*2*g*^ in position 27, operator *Â*_1*u*1*u*_^2*u*2*u*^ becomes one of the most significant contributors to the ansatz. The cooperative effect between operators *Â*_1*u*1*u*_^2*u*2*u*^ and *Â*_1*g*1*g*_^2*g*2*g*^ is evident when evaluating the energy without operator *Â*_1*u*1*u*_^2*u*2*u*^, which leads to an increase of 6.2 mHa. This energy shift exceeds the threshold for chemical accuracy,^35^ and is more than 2 orders of magnitude greater than the change observed when removing the poorly selected operators *Â*_1*g*1*g*_^4*u*4*u*^ and *Â*′_1*g*1*u*_^4*g*4*u*^ (in positions 19 and 20), as discussed in Section 2.1. This distinction underscores the difference between truly redundant operators and those that, despite initially having small parameter values, play a crucial role by collectively unlocking key regions of the Hilbert space.

## 3 Pruned-ADAPT-VQE Algorithm

Motivated by the various mechanisms that introduce ineffective operators into the ADAPT-VQE ansatz, and recognizing the potential for ansatz compaction, leading to shorter circuits without compromising energy accuracy, we develop an automated algorithm to remove these unnecessary contributions. The proposed method systematically eliminates poorly selected operators, those with fading parameter values, and redundant operators arising from reordering, while preserving operators involved in cooperative interactions. To achieve this, we introduce a simple yet effective routine, which we call Pruned-ADAPT-VQE.

We recognize that the criterion for removing the *i*th operator from the ansatz must take into account both the magnitude of its parameter θ_*i*_ and its position within the ansatz. To formalize this, we introduce a decision factor *f*_*i*_ for each operator, defined as the product of two functions: One dependent on the operator’s parameter and the other on its position

2 $$f_{i}=F_{1}\left(\theta_{i}\right)F_{2}\left(x_{i}\right)$$

where *x*_*i*_ is the relative position of the operator in an ansatz with *N* operators, given by *x*_*i*_ = *i*/*N*. We use this decision factor evaluated over each operator at each ADAPT-VQE iteration to determine which one will be removed. This formulation ensures that both small-amplitude operators and those appearing earlier in the ansatz are systematically evaluated for potential removal.

Among the possible functions that assign a larger factor to operators with smaller absolute parameter values, we select the inverse of the squared amplitude, as it naturally disregards the sign of θ_*i*_ and strongly emphasizes operators with near-zero parameter values

3 $$F_{1}\left(\theta_{i}\right)=\frac{1}{\theta_{i}^{2}}$$

Testing with alternative functions (|θ_*i*_^–*n*^|, with *n* = 0, 1, 3, 4) did not yield any noticeable advantage or improvement over the choice in eq 3 (see discussion in Section S3 of the Supporting Information).

Additionally, we prioritize the removal of operators with small parameter values that appear early in the ansatz. This prevents the elimination of cooperative operators and accounts for the natural decrease in amplitude of newly added operators as the ansatz approaches convergence. To achieve this, we introduce a position-dependent function *F*_2_ that decays exponentially with the operator’s position within the ansatz

4 $$F_{2}\left(x_{i}\right)=e^{-\alpha x_{i}}$$

where α is a positive hyperparameter controlling the influence of position on the removal criterion. Choosing an appropriate value for α requires balancing competing effects: a very large α would give priority to the operators in the first positions, preventing the removal of the intermediate or later ones, while a value approaching zero would make the position irrelevant, potentially eliminating recent additions that still contribute to convergence. Through preliminary testing, we find that when α is too small, proper convergence is not achieved, as the latest operator added is always removed in each iteration due to having the smallest coefficient. To prevent this, the function must assign sufficient relevance to the position. For the studied cases, we found that beyond a certain threshold (α ≈ 10) the method provides a reasonable compromise, ensuring effective pruning while maintaining smooth ansatz optimization (see the discussion in Section S4 of the Supporting Information).

Figure 6 illustrates the set of {*f*_*i*_} values when applied to the final ansatz (with 69 excitation operators) of the linear H_4_ molecule. The operator with the highest factor and selected for potential removal corresponds to operator in position 13, identified as a poorly selected operator and appearing along a flat energy region in Figure 1a.

**Figure 6:**
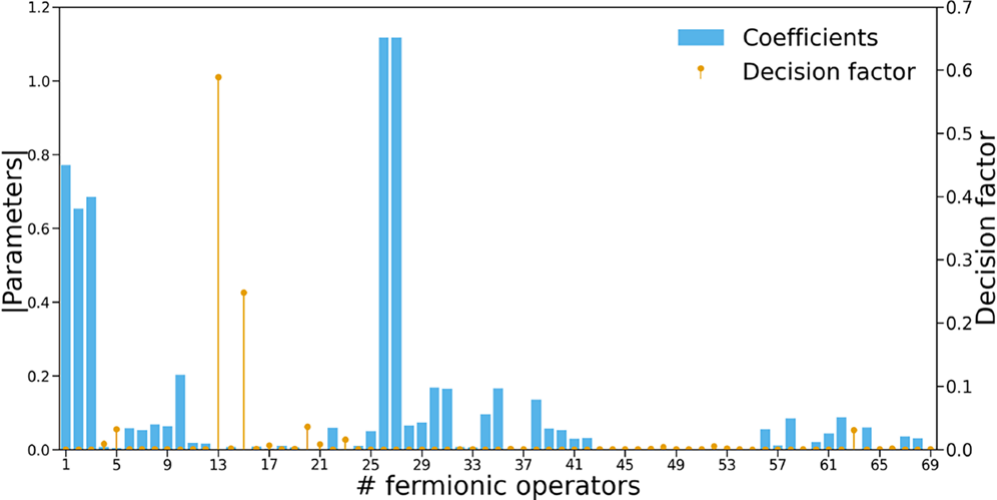
Absolute parameter values (blue bars) and decision factor values (orange sticks) for every ansatz operator of the linear H_4_ with interatomic distance of 3.0 Å, with the 3-21G basis set.

Note that, despite their proximity, operators 13 and 15 have coefficients on the order of 10^–4^, whereas operators 14 and 16–21 lie in the 10^–3^–10^–2^ range, leading to substantially lower decision factors. This distinction is not apparent in the plot, but the use of a logarithmic scale was avoided to ensure consistency with the rest of plots.

Therefore, to refine the ADAPT-VQE ansatz, we introduce a filtering process (Algorithm 1) that evaluates operators after each optimization iteration, identifying candidates for removal while preserving essential contributions. This approach prioritizes the removal of early appearing operators with negligibly small parameter values while retaining recently added operators that may play a cooperative role. To define τ, we use a fraction (0.1) of the average amplitude of the *N*_*L*_ most recently added operators

5 $$\tau =\frac{0.1}{N_{L}}\sum_{i=0}^{N_{L}-1}\left|\theta_{N-i}\right|$$

We examined the dependence of the dynamic threshold on *N*_*L*_ in eq 5, varying *N*_*L*_ from 1 to 10 (see Section S5 of the Supporting Information). Our analysis shows that adjusting this parameter has a mild impact on the final outcome for 2 ≤ *N*_*L*_ ≤ 10. Therefore, as a balanced choice, we set *N*_*L*_ = 4 for all subsequent analyses. We have also found (see Supporting Information Sections S5 and S9) that varying the fraction of the average amplitude provides a convenient way to control simulation convergence. Using a large fraction results in a more aggressive removal criterion and faster convergence, which can lead to premature termination if the fraction is too large. Conversely, a small fraction causes the simulation to behave more like the standard ADAPT-VQE.

The removal process is performed after a standard ADAPT-VQE iteration, as outlined in Algorithm 1, and proceeds without reoptimizing parameters, as the elimination of these low-impact operators has a negligible effect on the ansatz. As a result, this step does not increase the overall computational cost of the algorithm, as the evaluation of the decision factor and dynamic threshold is negligible. Figure S44 in Section S13 of the Supporting Information examines whether the removal of operators results in additional optimizer iterations in the classical routine, revealing a minor overhead that is mitigated by the end of the simulation. The Pruned-ADAPT-VQE routine (Algorithm 1) proceed as follows: (i) after each ADAPT-VQE iteration all operators in the ansatz are evaluated using eq 2; (ii) the parameter θ_*j*_ associated with the operator with the largest decision factor *f*_*j*_ is compared against a dynamic threshold τ, proceeding to the removal only if θ_*j*_ < τ; and (iii) the energy is calculated with the corresponding ansatz.
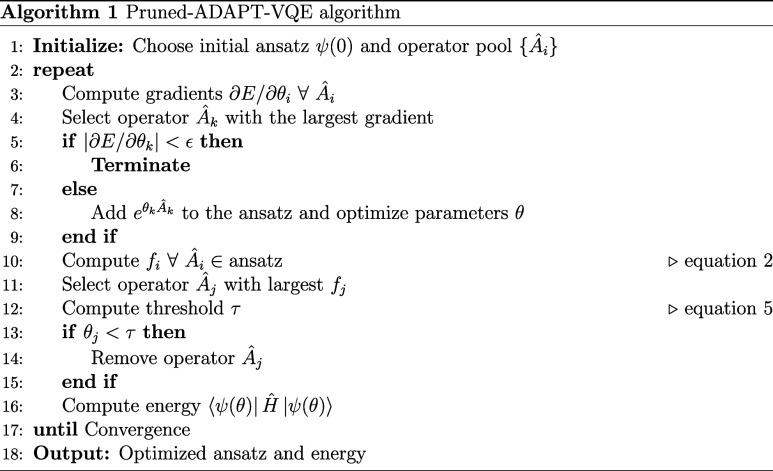


## 4 Performance of Pruned-ADAPT-VQE

Figure 7a compares the performance of Pruned-ADAPT-VQE against standard ADAPT-VQE in the simulation of linear H_4_. Without pruning, approximately 35 operators are needed to achieve chemical accuracy, whereas the refinement method reduces this number to 26. Initially, both approaches follow the same energy error profile. The pruning mechanism becomes active just after the flat energy region, around the 26th ADAPT iteration, ensuring that only noncooperative, unnecessary operators are removed while maintaining accuracy.

**Figure 7:**
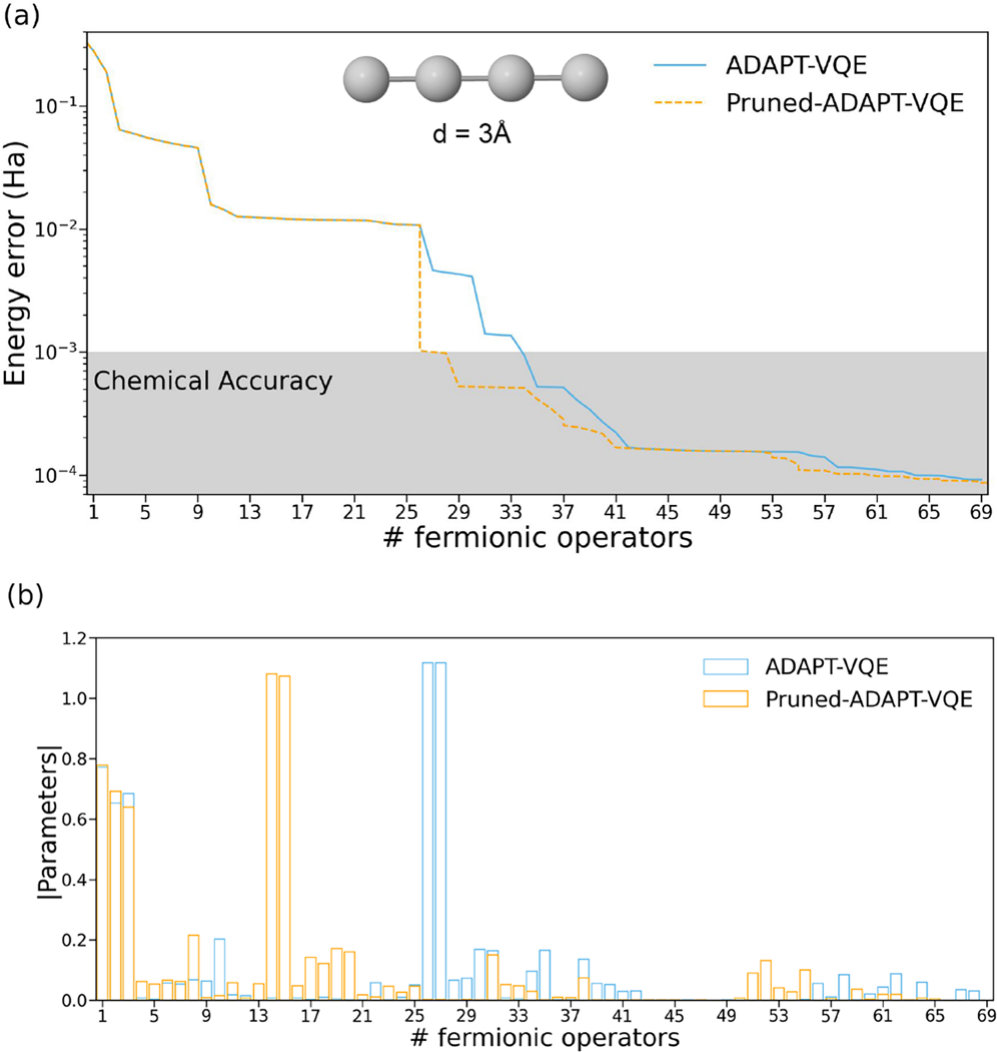
(a) Energy errors (in Hartree) with respect to FCI and (b) absolute parameter values for the *N* = 69 ansatz obtained with ADAPT-VQE (blue) and Pruned-ADAPT-VQE (orange) for the linear H_4_ system with interatomic distance of 3.0 Å and with the 3-21G basis set.

The ansatz compaction achieved through operator removal becomes evident when comparing the amplitude distributions of Pruned-ADAPT-VQE and standard ADAPT-VQE (Figure 7b). The elimination of irrelevant operators leads to a more compact ansatz, in which the most significant operators act earliest on the initial state. Our pruning strategy appears to be conservative, as several operators with near-zero parameter values remain. Adjusting the parameters that define the decision factor (eq 2) and the dynamic threshold (eq 5) could enable further (or more restrained) ansatz simplification, depending on computational constraints and accuracy requirements. For instance, increasing the 0.1 prefactor in the dynamic threshold enhances the likelihood of removing low-contributing operators, leading to more compact ansätze. While this may require additional ADAPT-VQE iterations to reach convergence, the trade-off can be favorable in scenarios where reducing circuit depth is critical due to hardware constraints. In fact, from length 53 to the end of the simulation, all removed operators were readded in the immediate next iteration, resulting in a significant computational overhead. Such behavior tends to appear toward the end of the simulation, when the ansatz is converging and the parameters associated with the added operators become vanishingly small. This behavior also becomes more pronounced in cases where the pruning strategy is more aggressive.

A closer examination of the operators removed throughout the Pruned-ADAPT-VQE iterations reveals distinct cases within the category of operators with small-valued parameter. The pruning strategy successfully eliminates wrongly selected excitations, such as operators *Â*_1*g*1*g*_^4*u*4*u*^ and *Â*′_1*g*1*u*_^4*g*4*u*^ (Figure 2), as well as fading operators like *Â*_1*g*1*u*_^2*g*4*u*^ and *Â*_1*g*1*u*_^4*g*2*u*^ (Figure 4). Additionally, it removes redundant operators arising from reordering, such as operator *Â*_1*g*_^3*g*^, which was removed and reinserted several times before finding a more suitable position that balances energy accuracy and ansatz length (Figure 3). Conversely, the algorithm effectively identifies and preserves cooperative operators, such as *Â*_1*u*1*u*_^2*u*2*u*^ (Figure 5), ensuring that essential contributions to the ansatz remain intact.

Similar results, demonstrating a reduction in the number of Fermionic operators without any loss of accuracy, have been observed in simulations of other molecular ground states. This includes the stretched H_2_O and N_2_ molecules (Figure 8) as well as in other systems (see Supporting Information, Section S8). As seen in the case of linear H_4_, ansatz reductions due to operator pruning tend to occur after flat energy regions in the ADAPT iterations. In these examples a computational overhead reappears toward the final simulation steps. For instance, in the H_2_O simulation, 5 operators are removed only to be reintroduced in the next iteration.

**Figure 8:**
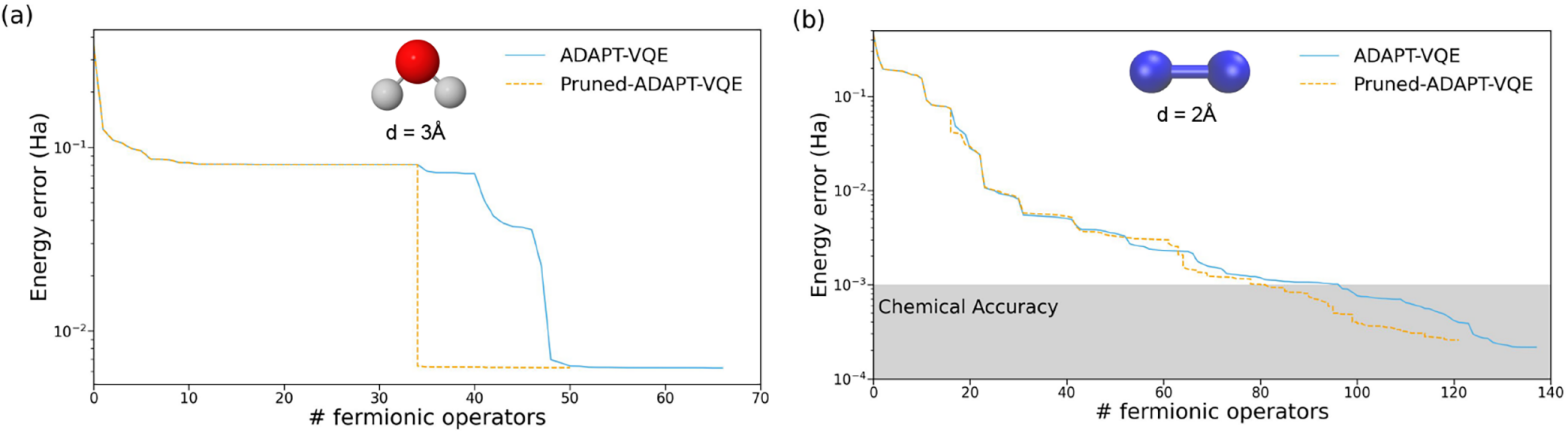
Energy errors (in Hartree) with respect to CAS­(8,8) (H_2_O) and CAS­(10,8) (N_2_) obtained with ADAPT-VQE (solid blue) and Pruned-ADAPT-VQE (dashed orange) ansätze for the (a) H_2_O with 3.0 Å O–H distance and computed in the 3-21G basis set with active space of 8 orbitals plus one frozen orbital, and (b) N_2_ with interatomic distance of 2.0 Å, and computed with the 3-21G basis set with 8 active plus 2 frozen orbitals.

It is worth noting that, in some cases, Pruned-ADAPT-VQE does not offer a substantial improvement over standard ADAPT-VQE in terms of ansatz reduction. For example, when computing ground-state energies using the minimal STO-3G basis set, the size of the operator pool is significantly reduced (see Supporting Information, Section S10), and Pruned-ADAPT-VQE exhibits a convergence profile nearly identical to that of ADAPT-VQE. These observations suggest that the benefits of pruning become more relevant when larger operator pools are involved, as typically encountered with larger basis sets in more realistic simulations. Additional comparisons of Pruned-ADAPT-VQE and ADAPT-VQE using various operator pools are presented in Supporting Information, Section S9. Notably, even in cases where operator pruning does not yield substantial ansatz reductions, the removal of excitation operators using the proposed approach never compromises the accuracy achieved by ADAPT-VQE.

## 5 Conclusions

The adaptive nature of ADAPT-VQE allows the ansatz to iteratively evolve, refining itself to better capture the problem landscape as the algorithm progresses. While the gradient-driven approach for ansatz growth offers a clear advantage over other strategies, it is not infallible. In some cases, the wave function may contain noncontributing operators, those with near-zero parameter values, which unnecessarily increase circuit depth without improving accuracy. In this work, we have identified three distinct mechanisms responsible for the presence of such redundant operators: (i) suboptimal operator selection due to limitations in the gradient criterion, (ii) operator reordering effects, where previously selected operators are reintroduced while the parameter values of their earlier instances diminish, leading to unnecessary redundancy in the ansatz, and (iii) fading operators, which become irrelevant as the ansatz evolves. Recognizing and addressing these issues enables the simplification and compression of ADAPT-VQE ansätze, leading to shorter quantum circuits without compromising accuracy.

Our approach systematically evaluates all ansatz operators after each optimization step, using a function that accounts for both operator position and amplitude. This strategy removes operators with minimal contributions while preserving those that may have cooperative effects in future iterations, which results in shallower circuits better suited to current NISQ hardware. By integrating this selection function with a dynamic threshold, adapted based on the parameter values of recently added operators, we effectively eliminate redundancies while ensuring algorithmic convergence. We validated this approach by computing the ground-state energies of several molecular systems. Our results demonstrate that this method potentially improves the efficiency of ADAPT-VQE simulations, particularly in scenarios with long energy plateaus, at the expense of a greater number of iterations. While in some instances the method does not lead to significant improvements, it consistently performs at least as well as standard ADAPT-VQE. Given its benefits in reducing ansatz complexity without introducing additional quantum computational overhead, we propose that this pruning strategy to be incorporated into ADAPT-VQE when circuit compactness is prioritized over iteration minimization.

## Supplementary Material



## Data Availability

The data that supports the findings of this study are available within the article and its Supporting Information. The code developed for the numerical simulations is openly available on GitHub.^29^

## Notes

Theory and methodology are provided; these include the definition of the notation of the Fermionic excitations in the pool (Section S1); and a description of the molecular orbitals of linear H_4_ (Section S2); details of both *F*_1_(θ_*i*_) (Section S3); and *F*_2_(*x*_*i*_) (Section S4) functions, part of the decision factor, are provided, as well as from the dynamical threshold (Section S5); analysis of the Pruned-ADAPT-VQE performance tested on several molecules (Section S6); analysis of the gradient-based selection criterion (Section S7); performance of Pruned-ADAPT-VQE with UCCSD (Section S8) and qubit-excitation-based (Section S9) pools; results with the STO-3G basis set (Section S10); dependence of parameter values (Section S11); gate counts (Section S12) (PDF)
