# Supplementary material for: Pruned-ADAPT-VQE: Compacting Molecular Ansätze by Removing Irrelevant Operators
Source: J Chem Theory Comput. 2025 Sep 6;21(18):8720–8. doi: 10.1021/acs.jctc.5c00535 (PMC12461939; doi:10.1021/acs.jctc.5c00535)
Supplement: Supplementary file 1 [file ct5c00535_si_001.pdf]

Supporting Information: Pruned-ADAPT-VQE: compacting molecular ansätze by removing irrelevant operators

Nonia Vaquero-Sabater,[†,‡] Abel Carreras,[*,†] and David Casanova[*,†,¶]

†*Donostia International Physics Center (DIPC), 20018 Donostia, Euskadi, Spain*

‡*Polimero eta Material Aurreratuak: Fisika, Kimika eta Teknologia Saila, Kimika Fakultatea, Euskal Herriko Unibertsitatea (EHU), PK 1072, 20080 Donostia, Euskadi, Spain*

¶*IKERBASQUE, Basque Foundation for Science, 48009 Bilbao, Euskadi, Spain*

E-mail: abelcarreras83@gmail.com; david.casanova@dipc.org

Contents

S1 Excitation operators in the pool **S-3**

S2 Molecular orbitals of H_4 and H_2O **S-4**

S3 Alternative functions for $F_1(\theta_i)$ **S-6**

S4 Dependence on α value in $F_2(x_i)$ **S-8**

S5 Threshold analysis **S-10**

S6 Additional molecule analysis **S-13**

S7 The gradient-based selection criterion **S-16**

S8 Pruned-ADAPT-VQE with UCCSD pool **S-17**

S9 Pruned-ADAPT-VQE with qubit-excitation-based pool **S-20**

S10 STO-3G basis testing **S-24**

S10.1 Fermionic pool S-25

S10.2 Qubit-excitation-based pool S-26

S11 Energy difference vs parameter value **S-27**

S12 Gate counts **S-28**

S13 Impact of Removal on Classical Optimization Cost **S-30**

References **S-31**

S1 Excitation operators in the pool

Each individual excitation operator in the pool consists of a UCC operator restricted to occupied-to-virtual spin-singlet adapted single and double excitations. Occupied (virtual) orbitals in the HF determinant are indicated with i, j (a, b) indices. Single and double excitations take the form:

$$\hat{A}_i^a = \frac{1}{2}\left[\hat{\tau}_i^a + \hat{\tau}_{\bar{i}}^{\bar{a}} - \text{h.c.}\right] \tag{S1}$$

$$\hat{A}_{ii}^{aa} = \frac{1}{\sqrt{2}}\left[\hat{\tau}_{i\bar{i}}^{a\bar{a}} - \text{h.c.}\right] \tag{S2}$$

$$\hat{A}_{ii}^{ab} = \frac{1}{2}\left[\hat{\tau}_{i\bar{i}}^{a\bar{b}} + \hat{\tau}_{i\bar{i}}^{\bar{a}b} - \text{h.c.}\right] \tag{S3}$$

$$\hat{A}_{ij}^{aa} = \frac{1}{2}\left[\hat{\tau}_{i\bar{j}}^{a\bar{a}} + \hat{\tau}_{\bar{i}j}^{\bar{a}a} - \text{h.c.}\right] \tag{S4}$$

$$\hat{A}_{ij}^{ab} = \frac{1}{2\sqrt{6}}\left[2(\hat{\tau}_{ij}^{ab} + \hat{\tau}_{\bar{i}\bar{j}}^{\bar{a}\bar{b}}) + \hat{\tau}_{i\bar{j}}^{a\bar{b}} + \hat{\tau}_{\bar{i}j}^{\bar{a}b} + \hat{\tau}_{\bar{i}j}^{a\bar{b}} + \hat{\tau}_{i\bar{j}}^{\bar{a}b} - \text{h.c.}\right] \tag{S5}$$

$$\hat{A'}_{ij}^{ab} = \frac{1}{2\sqrt{2}}\left[\hat{\tau}_{i\bar{j}}^{a\bar{b}} + \hat{\tau}_{\bar{i}j}^{\bar{a}b} - \hat{\tau}_{\bar{i}j}^{a\bar{b}} - \hat{\tau}_{i\bar{j}}^{\bar{a}b} - \text{h.c.}\right] \tag{S6}$$

(S7)

where h.c. indicates hermitian conjugate, the bar over orbital indices refer to β-spin orbitals, and $\hat{\tau}_i^a$ and $\hat{\tau}_{ij}^{ab}$ are expressed in terms of creation and annihilation operators as:

$$\hat{\tau}_i^a = \hat{a}_a^\dagger \hat{a}_i \tag{S8}$$

$$\hat{\tau}_{ij}^{ab} = \hat{a}_a^\dagger \hat{a}_b^\dagger \hat{a}_j \hat{a}_i \tag{S9}$$

S2 Molecular orbitals of H_4 and H_2O

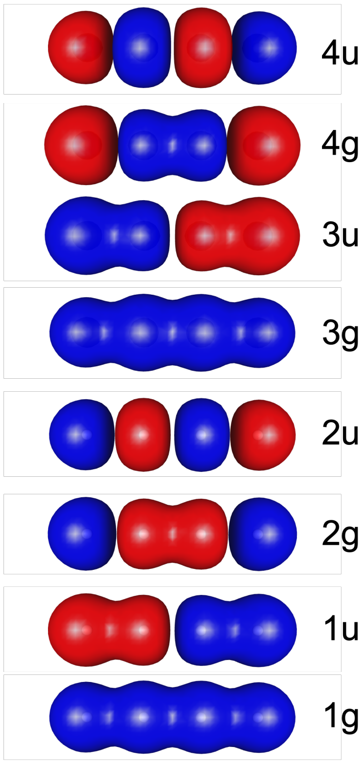

Figure S1: Molecular orbitals computed at the HF/3-21G level of linear H_4 with 3.0 Å intermolecular separation

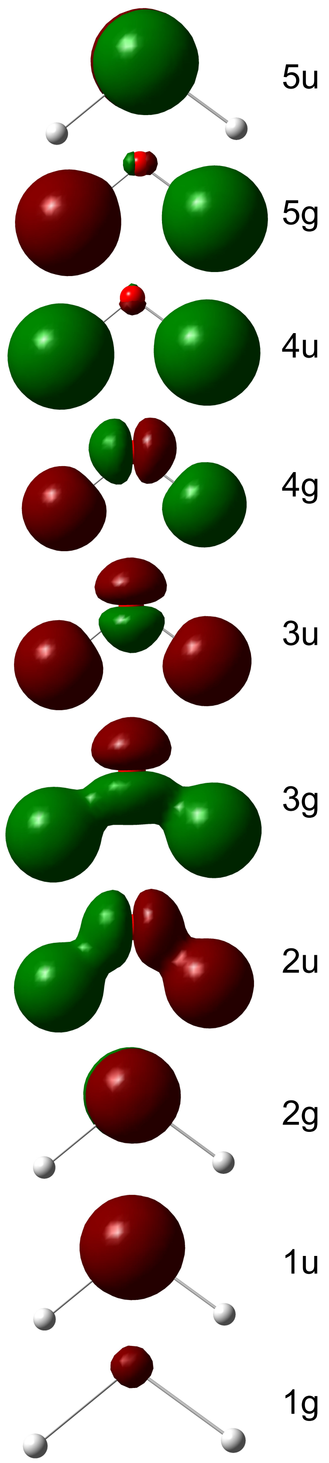

Figure S2: First 10 molecular orbitals computed at the HF/3-21G level of H_2O with 3.0 Å intermolecular H−O separation

In the orbital labels, the subscripts g (gerade) and u (ungerade) denote the symmetry of the molecular orbitals with respect to inversion through the center of mass. A gerade orbital is symmetric under inversion (i.e., its wavefunction remains unchanged), while an ungerade orbital is antisymmetric (i.e., the wavefunction changes its sign).

S3 Alternative functions for $F_1(\theta_i)$

The objective of this term is to account for the amplitude of each operator, prioritizing those with the smallest contributions. In this section, several values of n ($n = 0, 1, 3, 4$) in equation S10 have been tested.

$$F_1(\theta_i) = \frac{1}{|\theta_i^{-n}|} \tag{S10}$$

Each value of n represents a different weight assigned to the amplitude within the total function.

θ_i^{-0} means that no weight is assigned to the amplitude, giving no preference to operators with small coefficients. This function is expected to yield the same result as ADAPT-VQE. On the other hand, θ_i^{-4} assigns a very high weight to the amplitude. While the position is still considered, the influence of the amplitude becomes dominant, leading to the selection of operators with the smallest coefficients over those in earlier positions. Since operator amplitudes tend to decrease as the simulation progresses, this would result in the last operator being repeatedly selected, preventing the simulation from converging (see $n = 4$ in Figure S3 and $n = 3, 4$ in Figures S4 and S5).

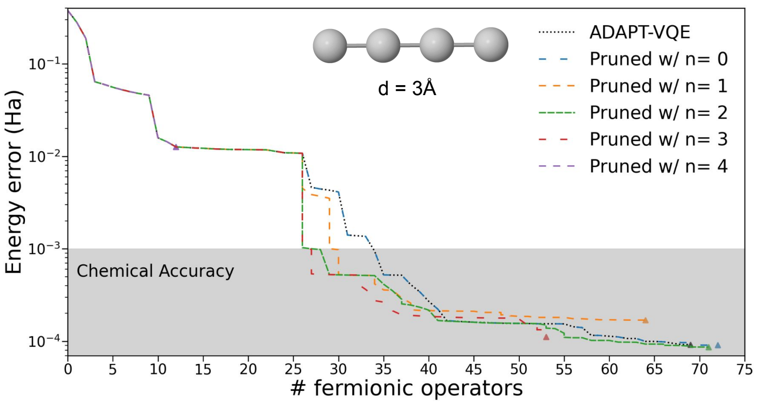

Figure S3: Energy error (in Hartree) with respect to FCI of ADAPT-VQE (black dotted line) and Pruned-ADAPT-VQE with different amplitude weights for the linear H_4 with interatomic distance of 3.0 Å, and computed with the 3-21G basis set and an active space of 8 orbitals. The triangle indicates where the simulation ended.

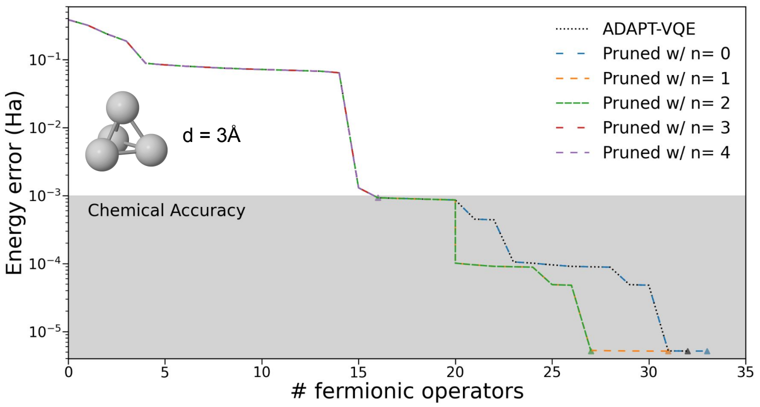

Figure S4: Energy error (in Hartree) with respect to FCI of ADAPT-VQE (black dotted line) and Pruned-ADAPT-VQE with different amplitude weights for the tetrahedral H_4 with interatomic distance of 3.0 Å, and computed with the 3-21G basis set and an active space of 8 orbitals. The triangle indicates where the simulation ended.

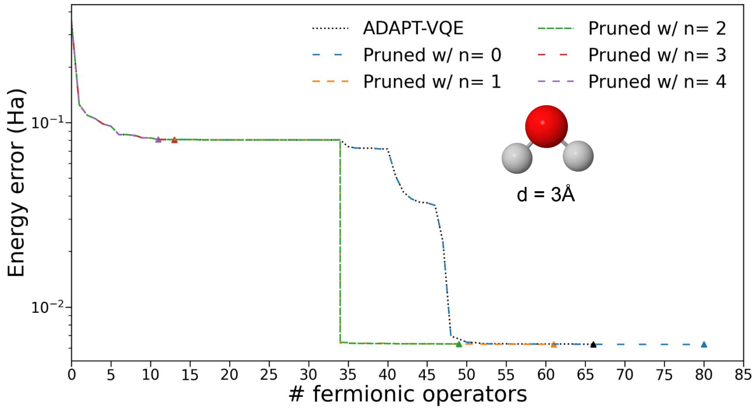

Figure S5: Energy error (in Hartree) with respect to CAS(8,8) of ADAPT-VQE (black dotted line) and Pruned-ADAPT-VQE with different amplitude weights for the H_2O molecule with 3.0 Å O−H distance, and computed with the 3-21G basis set with active space of 8 orbitals plus one frozen orbital. The triangle indicates where the simulation ended.

S4 Dependence on α value in $F_2(x_i)$

The objective of this term is to take into account the position of each operator. The weight of the position is modulated by the α value in equation S11.

$$F_2(x_i) = e^{-\alpha x_i} \tag{S11}$$

Setting $\alpha = 0$ gives no importance to position, meaning the total function would depend only on the coefficient. This would cause the latter operators to always be removed, as the algorithm tends to add operators with smaller coefficients over time. A high value of α may overly prioritize the operators at the beginning of the ansatz, potentially preventing some intermediate operators from being removed. This effect can be visualized in Figures S6, S7, and S8, where the smallest α values make the simulation end prematurely while the biggest ones lead to longer ansätze.

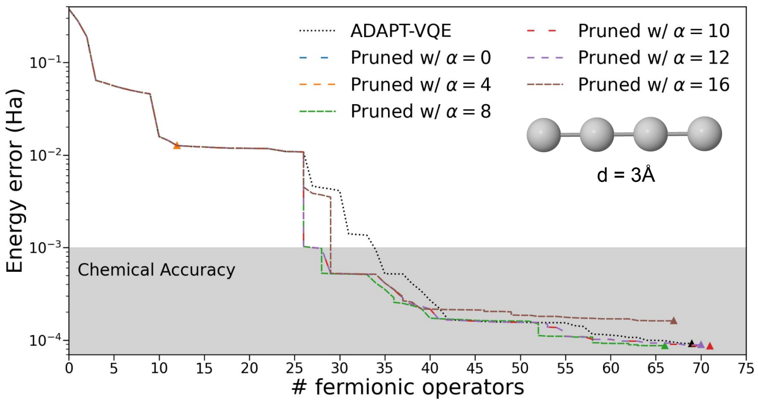

Figure S6: Energy error (in Hartree) with respect to FCI of ADAPT-VQE (black dotted line) and Pruned-ADAPT-VQE with different α values for the linear H_4 with interatomic distance of 3.0 Å, and computed with the 3-21G basis set and an active space of 8 orbitals. The triangle indicates where the simulation ended.

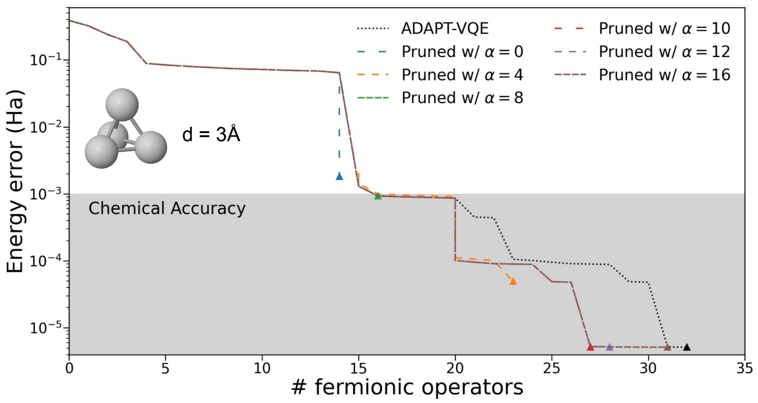

Figure S7: Energy error (in Hartree) with respect to FCI of ADAPT-VQE (black dotted line) and Pruned-ADAPT-VQE with different α values for the tetrahedral H_4 with interatomic distance of 3.0 Å, and computed with the 3-21G basis set and an active space of 8 orbitals. The triangle indicates where the simulation ended.

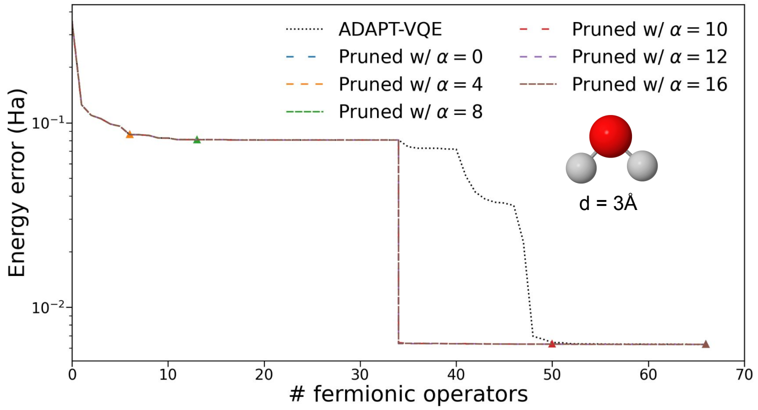

Figure S8: Energy error (in Hartree) with respect to CAS(8,8) of ADAPT-VQE (black dotted line) and Pruned-ADAPT-VQE with α values weights for the H_2O molecule with 3.0 Å O−H distance, and computed with the 3-21G basis set with active space of 8 orbitals plus one frozen orbital. The triangle indicates where the simulation ended.

S5 Threshold analysis

Once an operator is selected using the decision factor, we must determine whether to remove it based on a threshold. As the simulation progresses, amplitudes tend to decrease, so operators with smaller coefficients must be allowed into the ansatz, this is taken into account with a dynamic threshold. Equation S12 shows that the threshold value depends on the coefficients of the most N_L recently added operators in the ansatz.

$$\tau = \frac{0.1}{N_L} \sum_{i=0}^{N_L - 1} |\theta_{N-i}| \tag{S12}$$

where N is to total number of operators in the ansatz. This adaptive approach facilitates convergence, as the decreasing coefficients lead to a progressively lower threshold, resulting in fewer operators being removed over time. Additionally, it enables the elimination of operators in flat regions, where subsequent operators tend to have larger amplitudes, causing the threshold to rise. Several tests have been conducted, considering the last N_L operators, with N_L ranging from 1 to 10.

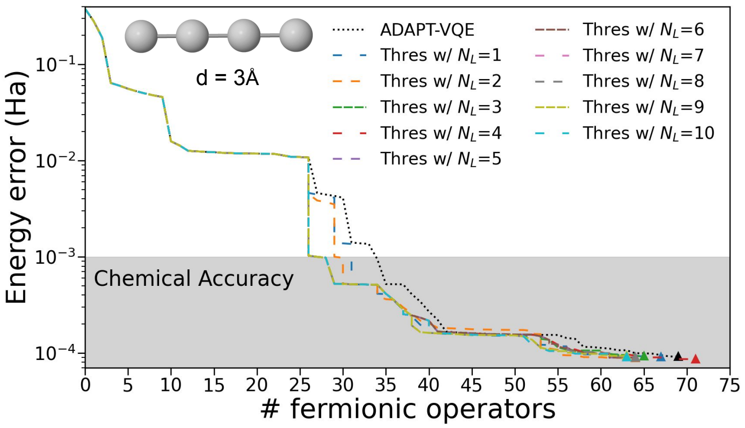

Figure S9: Energy error (in Hartree) with respect to FCI of ADAPT-VQE (black dotted line) and Pruned-ADAPT-VQE with different N_L values for the linear H_4 with interatomic distance of 3.0 Å, and computed with the 3-21G basis set and an active space of 8 orbitals. The triangle indicates where the simulation ended.

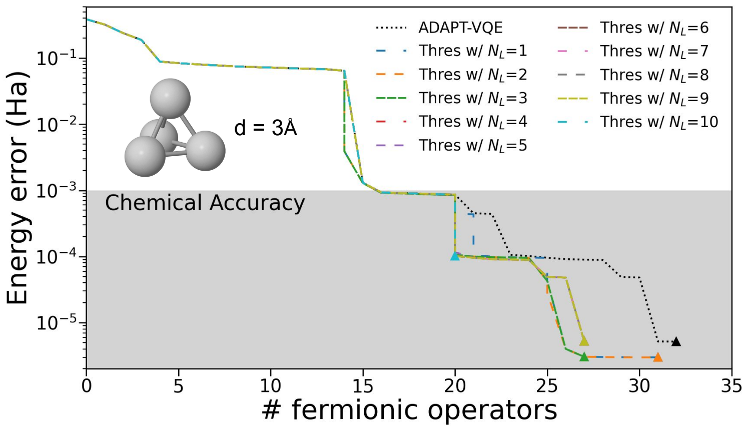

Figure S10: Energy error (in Hartree) with respect to FCI of ADAPT-VQE (black dotted line) and Pruned-ADAPT-VQE with different N_L values for the tetrahedral H_4 with interatomic distance of 3.0 Å, and computed with the 3-21G basis set and an active space of 8 orbitals. The triangle indicates where the simulation ended.

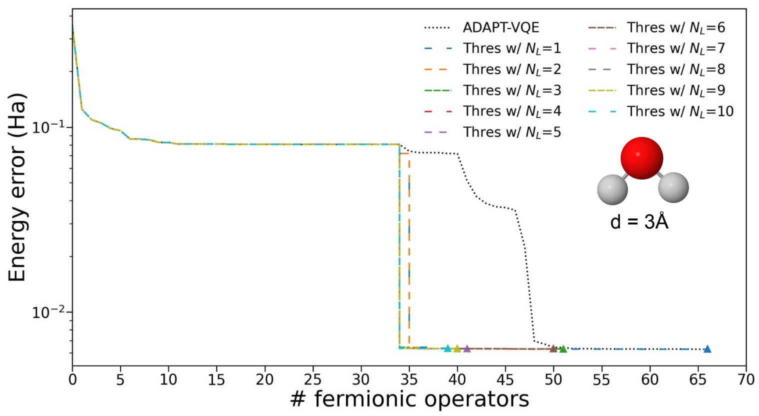

Figure S11: Energy error (in Hartree) with respect to CASCI(8,8) of ADAPT-VQE (black dotted line) and Pruned-ADAPT-VQE with N_L values weights for the H_2O molecule with 3.0 Å O−H distance, and computed with the 3-21G basis set with active space of 8 orbitals plus one frozen orbital. The triangle indicates where the simulation ended.

Setting the prefactor to 0.1 in equation S12 balances the likelihood of removing low-contributing operators. Decreasing it allow smaller operators appear in the ansatz and reach smaller errors, while increasing it creates more compact ansätze that may not reach the lowest energies. A large prefactor can also trigger premature operator removals, causing operators to be reintroduced in subsequent iterations and resulting in non-useful iterations. Figures S12-S14 study this effect by varying the prefactor we denominate from 0.2 to 0.01.

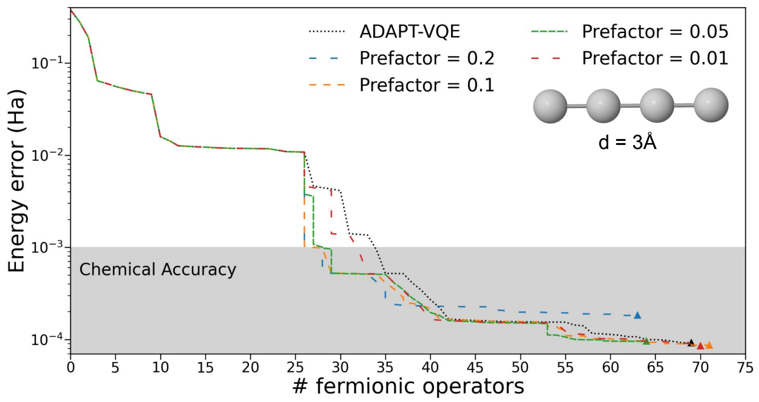

Figure S12: Energy error (in Hartree) with respect to FCI of ADAPT-VQE (black dotted line) and Pruned-ADAPT-VQE with different prefactor values for the linear H_4 with inter-atomic distance of 3.0 Å, and computed with the 3-21G basis set and an active space of 8 orbitals. The triangle indicates where the simulation ended.

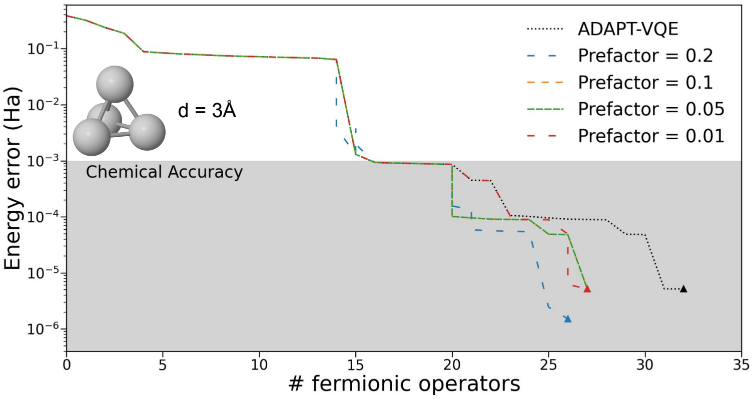

Figure S13: Energy error (in Hartree) with respect to FCI of ADAPT-VQE (black dotted line) and Pruned-ADAPT-VQE with different prefactor values for the tetrahedral H_4 with interatomic distance of 3.0 Å, and computed with the 3-21G basis set and an active space of 8 orbitals. The triangle indicates where the simulation ended.

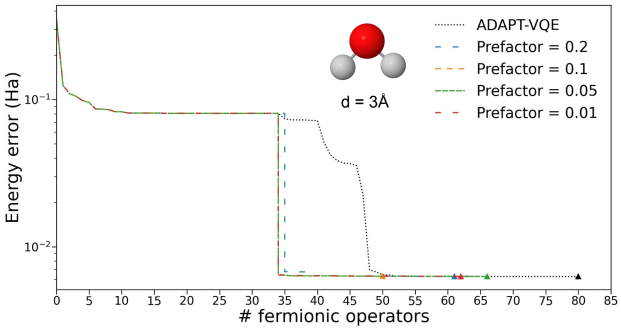

Figure S14: Energy error (in Hartree) with respect to CASCI(8,8) of ADAPT-VQE (black dotted line) and Pruned-ADAPT-VQE with different prefactor values for the H_2O molecule with 3.0 Å O−H distance, and computed with the 3-21G basis set with active space of 8 orbitals plus one frozen orbital. The triangle indicates where the simulation ended.

We conclude that setting the prefactor to 0.1 achieves, in general, a good compromise between accuracy and ansatz size. However, as observed in Figure S13, in some cases an aggressive removal criteria may lead to more efficient ansätze.

S6 Additional molecule analysis

In this section we analyze the removed operators from the H_2O molecule. We observe again the operator reordering phenomena in some of the removed operators. This is the case of

operator $\hat{A}_{3g}^{4u}$, Figure S15, where the addition of the same operator in iteration 43 causes a decrease in the parameter value of the first added operator, leading to its removal. The same happens with the second $\hat{A}_{3g}^{4u}$ added, whose coefficient decays after a third addition of the same instance. In this case, however, the weight is the weight is distributed between the two instances of the operator, and neither is excluded from the ansatz. This illustrates another instance of local quasi-commutation between the operator and this segment of the ansatz.

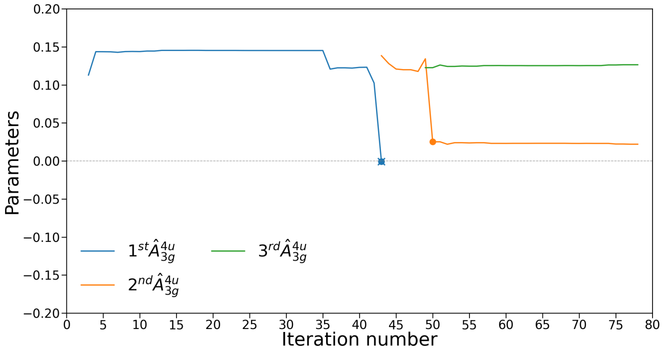

Figure S15: Parameter values of various operators from their introduction to iteration 80 in the simulation of H_2O with interatomic distance H−O of 3.0 Å and the 3-21G basis set, using an active space of 8 orbitals plus 1 frozen orbital. Full circles indicate introduction of another instance of the operator. Cross markers indicate when the operator is removed by Pruned-ADAPT-VQE.

Another example of operator reordering is $\hat{A}_{4g4g}^{1u1u}$, shown in Figure S16.

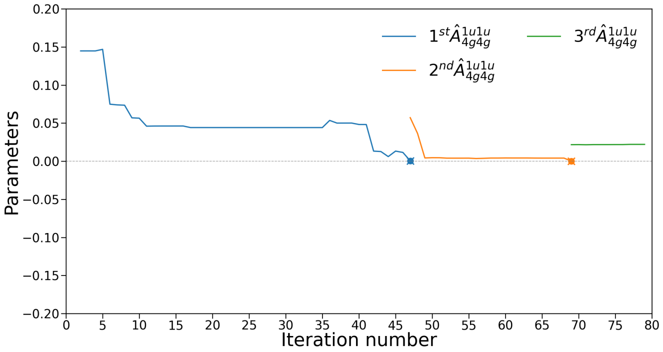

Figure S16: Parameter values of various operators from their introduction to iteration 80 in the simulation of H_2O with interatomic distance H−O of 3.0 Å and the 3-21G basis set, using an active space of 8 orbitals plus 1 frozen orbital. Full circles indicate introduction of another instance of the operator. Cross markers indicate when the operator is removed by Pruned-ADAPT-VQE.

Almost all of the deleted operators in this simulation are due to what we defined as bad operator selection. Some examples are shown in Figure S17.

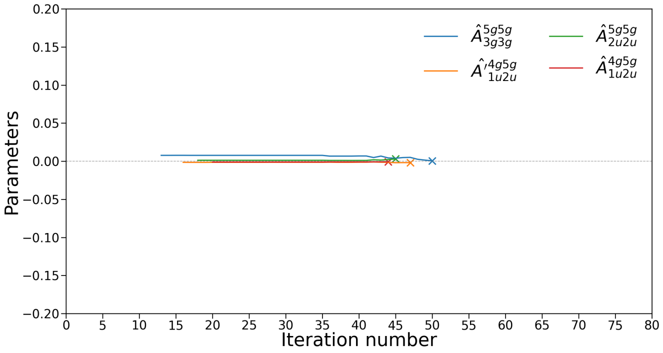

Figure S17: Parameter values of various operators from their introduction to iteration 80 in the simulation of H_2O with interatomic distance H−O of 3.0 Åand the 3-21G basis set, using an active space of 8 orbitals plus 1 frozen orbital. Full circles indicate introduction of another instance of the operator. Cross markers indicate when the operator is removed by Pruned-ADAPT-VQE.

The simulation results are shown in Figure 8, where a large flat area is observed. Figure S18 illustrates the evolution of the total gradient throughout the simulation, linking this flat region to a gradient trough. We attribute this phenomenon to the large number of poor operators that were selected during this simulation.

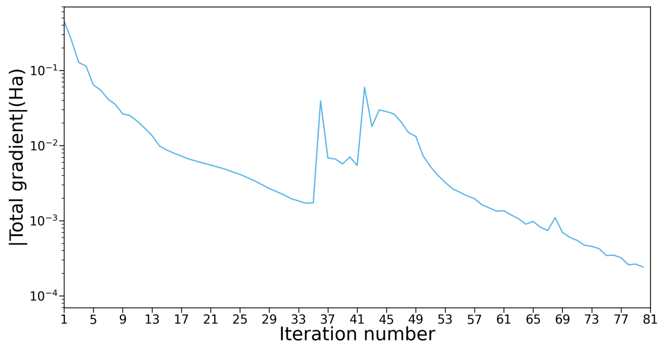

Figure S18: Total gradient of the UCCSD operators in ADAPT-VQE simulation of H_2O molecule with 3.0 Å O−H distance, and computed with the 3-21G basis set with active space of 8 orbitals plus one frozen orbital.

The comparison between the final ansätze of ADAPT-VQE and Pruned-ADAPT-VQE is shown in Figure S19.

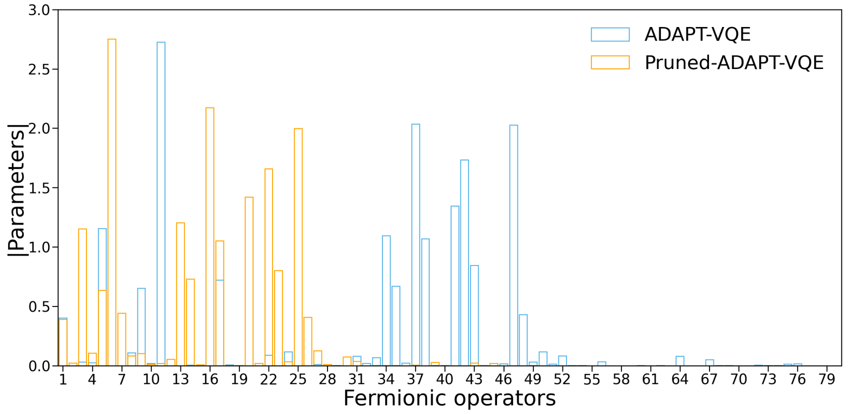

Figure S19: Absolute parameter values for the N = 80 ansatz obtained with ADAPT-VQE (blue) and Pruned-ADAPT-VQE (orange) for the H_2O system with interatomic distance H−O of 3.0 Å and with the 3-21G basis set using 8 active orbitals plus one frozen orbital.

S7 The gradient-based selection criterion

This section tests wether the operator with the largest gradient is always the best choice. We use the first 12 operators from the fermionic ADAPT-VQE ansatz for the linear H_4 system with interatomic distance of 3.0 Å (example from Figure 1). We take the first 12 operators

because a gradient trough appears starting from that point, as can be appreciated in Figure 1c, which leads to a sequence of iterations where the energy error barely improves, this is what we call flat area. After taking the 12-operators ansatz from ADAPT-VQE, instead of selecting the next one to be added with the gradient criteria, we try every possible operator in the pool and optimize all the parameters. Repeating this process three times, led to a different ansatz that was able to avoid the flat area, as is shown in Figure S20.

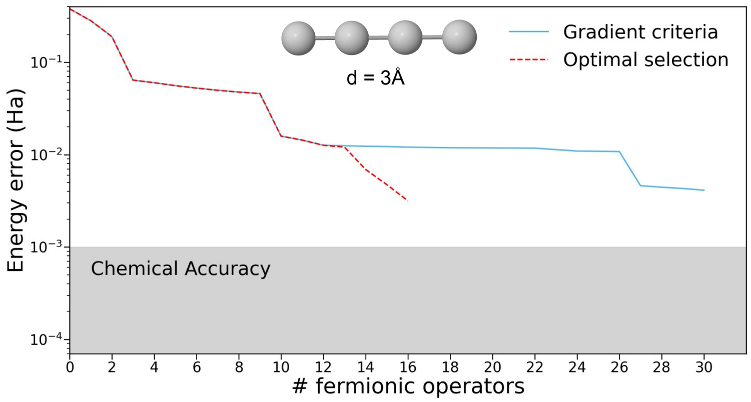

Figure S20: Energy error (in Hartree) with respect to FCI of ADAPT-VQE using the gradient-based criteria (blue line) and trying all possible options and keeping the best one (dotted red line) for the linear H_4 with interatomic distance of 3.0 Å, and computed with the 3-21G basis set and active space of 8 orbitals.

S8 Pruned-ADAPT-VQE with UCCSD pool

In this section we test Pruned-ADAPT-VQE on a variety of molecular systems. The hyper-parameters used are the same as those employed in the main text examples: a coefficient weight of 2, a prefactor $\alpha = 10$, and a dynamic threshold set to 10% of the average amplitude of the last four added operators.

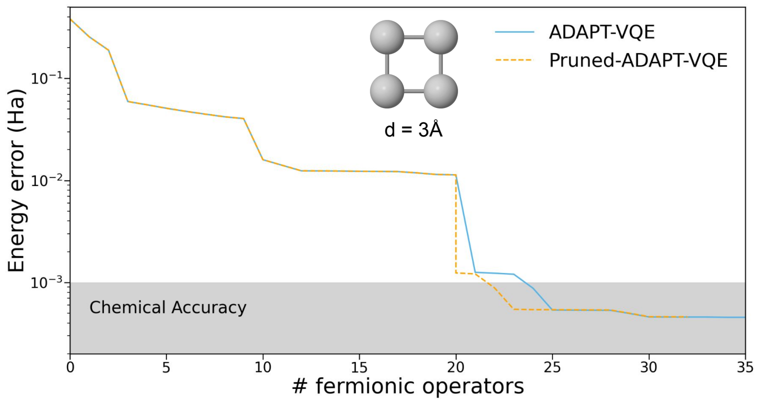

Figure S21: Energy errors (in Hartree) with respect to FCI obtained with ADAPT-VQE (solid blue) and Pruned-ADAPT-VQE (dashed orange) ansätze for the squared H_4 with interatomic distance of 3.0 Å, and computed with the 3-21G basis set and an active space of 8 orbitals.

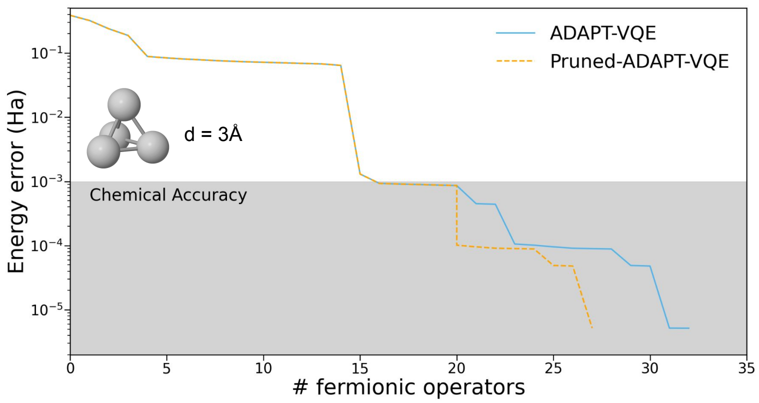

Figure S22: Energy errors (in Hartree) with respect to FCI obtained with ADAPT-VQE (solid blue) and Pruned-ADAPT-VQE (dashed orange) ansätze for the tetrahedral H_4 with interatomic distance of 3.0 Å, and computed with the 3-21G basis set and an active space of 8 orbitals.

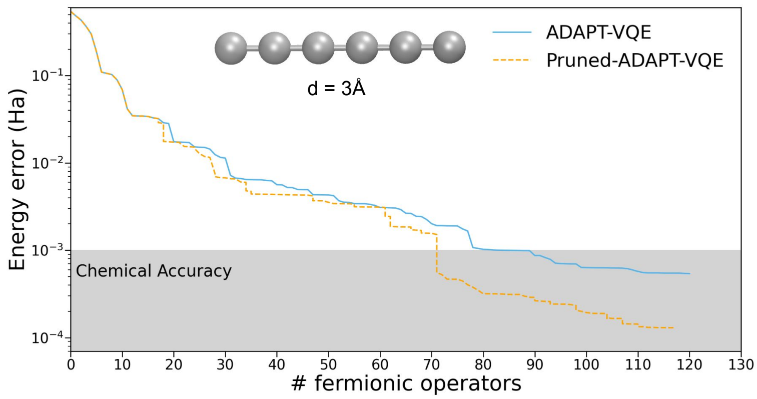

Figure S23: Energy errors (in Hartree) with respect to CASCI(6,8) obtained with ADAPT-VQE (solid blue) and Pruned-ADAPT-VQE (dashed orange) ansätze for the linear H_6 system with interatomic distance of 3.0 Å, and computed with the 3-21G basis set and an active space of 8 orbitals.

In the case of BeH_2 we found that using the same parameters led to a premature convergence of the algorithm. This situation was resolved by taking a more relaxed removal strategy, specifically by reducing the prefactor in the threshold definition.

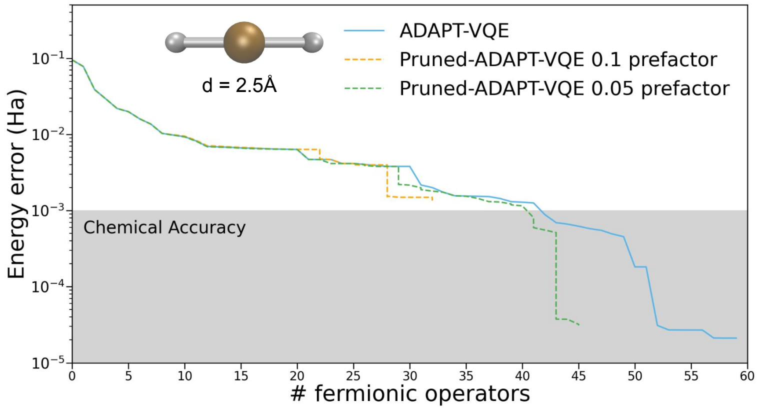

Figure S24: Energy errors (in Hartree) with respect to CASCI(4,8) obtained with ADAPT-VQE (solid blue) and Pruned-ADAPT-VQE (dashed orange for prefactor 0.1 and dashed green for prefactor 0.05, see Section 5) ansätze for the BeH_2 molecule with interatomic distance of 2.5 Å, and computed with the 3-21G basis set and an active space of 8 orbitals plus one frozen orbital.

S9 Pruned-ADAPT-VQE with qubit-excitation-based pool

In this section, we evaluate the performance of Pruned-ADAPT-VQE using the qubit-excitation-based pool.[S1] Unless otherwise specified, we employ the same hyperparameters as those used with the fermionic pool: a coefficient weight of 2, $\alpha = 10$, and a threshold defined as 10% of the average amplitude of the last 4 added operators.

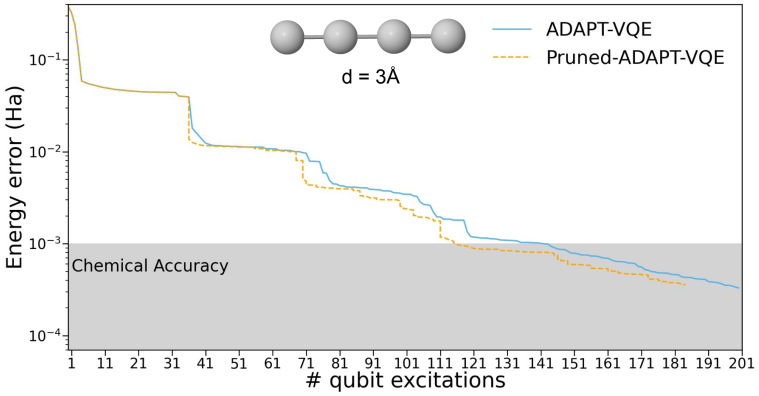

Figure S25: Energy errors (in Hartree) with respect to FCI obtained with ADAPT-VQE (solid blue) and Pruned-ADAPT-VQE (dashed orange) ansätze with qubit-excitation-based pool for the linear H_4 molecule with interatomic distance of 3.0 Å, and computed with the 3-21G basis set and an active space of 8 orbitals.

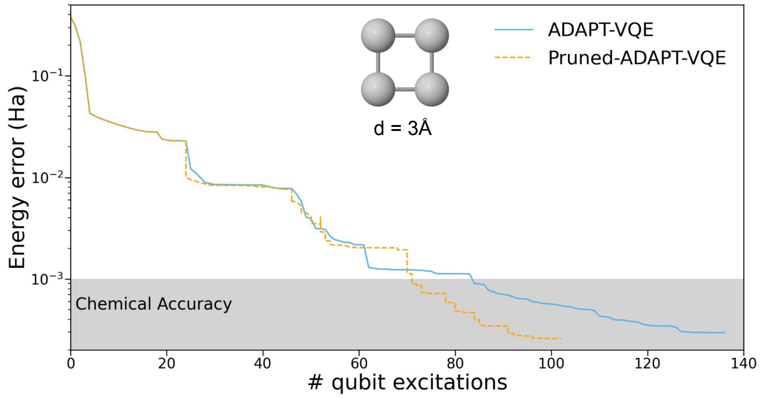

Figure S26: Energy errors (in Hartree) with respect to FCI obtained with ADAPT-VQE (solid blue) and Pruned-ADAPT-VQE (dashed orange) ansätze with qubit-excitation-based pool for the squared H_4 molecule with interatomic distance of 3.0 Å, and computed with the 3-21G basis set and an active space of 8 orbitals with one frozen orbital.

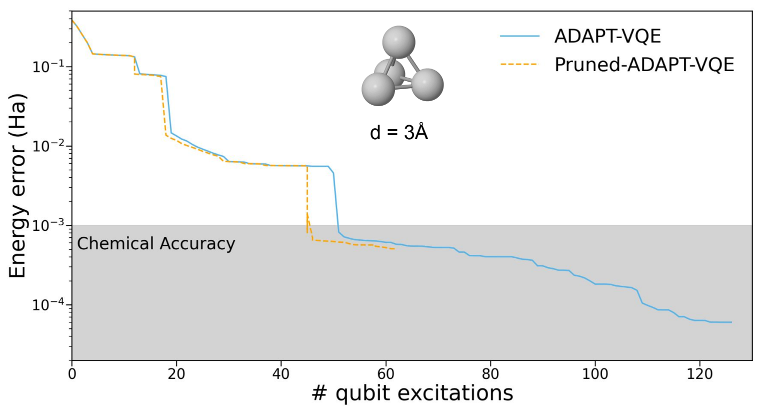

Figure S27: Energy errors (in Hartree) with respect to FCI obtained with ADAPT-VQE (solid blue) and Pruned-ADAPT-VQE (dashed orange) ansätze with qubit-excitation-based pool for the tetrahedral H_4 molecule with interatomic distance of 3.0 Å, and computed with the 3-21G basis set and an active space of 8 orbitals with one frozen orbital.

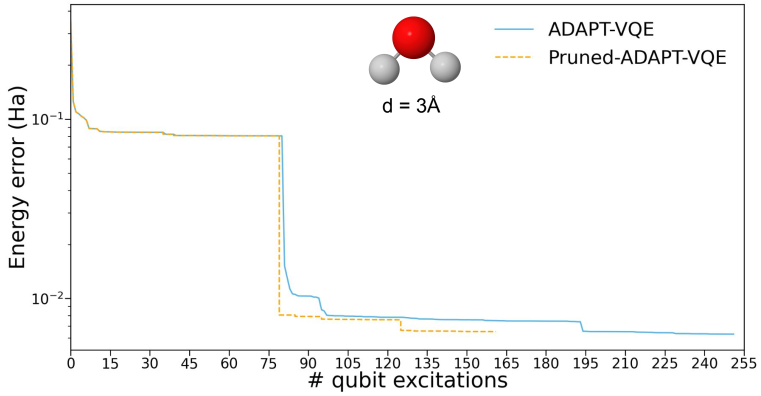

Figure S28: Energy errors (in Hartree) with respect to CASCI obtained with ADAPT-VQE (solid blue) and Pruned-ADAPT-VQE (dashed orange) ansätze with qubit-excitation-based pool for the H_2O with 3.0 Å O−H distance and computed in the 3-21G basis set with active space of 8 orbitals plus one frozen orbital.

In the case of the tetrahedral H_4 molecule, using a prefactor of 0.1 leads to premature convergence of the simulation. This suggest that in this case 0.1 may be a bit too aggressive. Decreasing the threshold allows the simulation to reach lower energy values, preventing the premature removal of relevant operators. In Figures S29-S31 we test different prefactor values for equation S12.

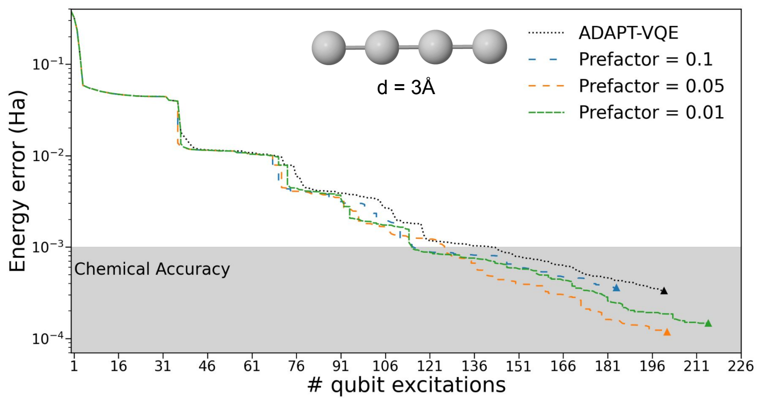

Figure S29: Energy errors (in Hartree) with respect to FCI obtained with ADAPT-VQE (solid blue) and Pruned-ADAPT-VQE (dashed orange) ansätze with qubit-excitation-based pool and different prefactor values for the linear H_4 molecule with interatomic distance of 3.0 Å, and computed with the 3-21G basis set and an active space of 8 orbitals.

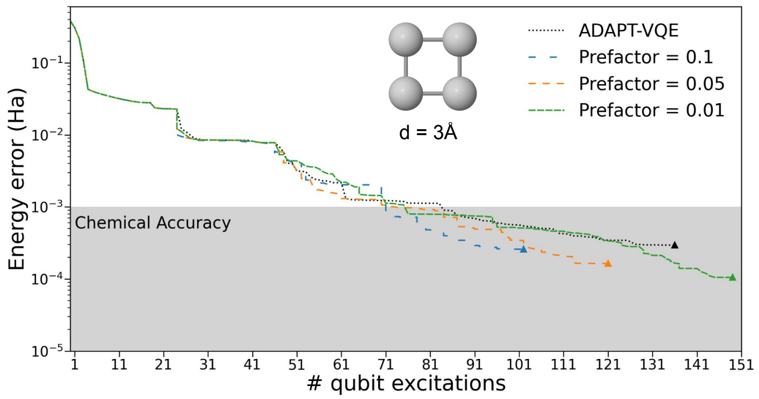

Figure S30: Energy errors (in Hartree) with respect to FCI obtained with ADAPT-VQE (solid blue) and Pruned-ADAPT-VQE (dashed orange) ansätze with qubit-excitation-based pool and different prefactor values for the squared H_4 molecule with interatomic distance of 3.0 Å, and computed with the 3-21G basis set and an active space of 8 orbitals.

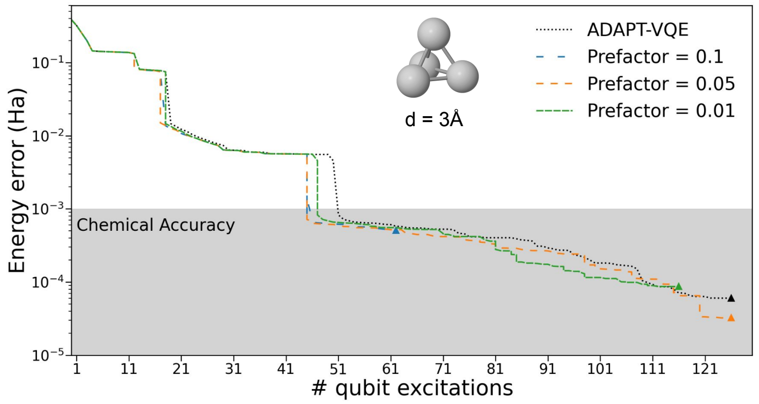

Figure S31: Energy errors (in Hartree) with respect to FCI obtained with ADAPT-VQE (solid blue) and Pruned-ADAPT-VQE (dashed orange) ansätze with qubit-excitation-based pool and different prefactor values for the tetrahedral H_4 molecule with interatomic distance of 3.0 Å, and computed with the 3-21G basis set and an active space of 8 orbitals.

We find that when using the qubit-excitation-based pool, setting the threshold prefactor to 5% of the average amplitude of the most recently added operators yields a better compromise between accuracy and circuit depth than the 10% value used in the main text. This suggests that the prefactor may need to be adjusted depending on both the target accuracy and the choice of operator pool. The example in Figure S32 have been carried out using a prefactor of 0.05.

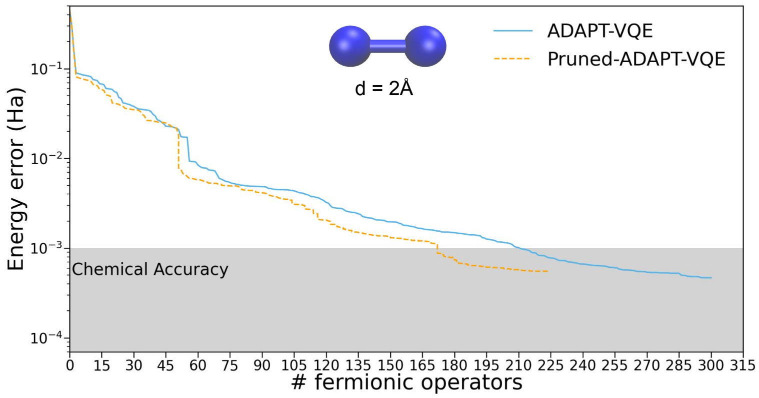

Figure S32: Energy errors (in Hartree) with respect to CASCI(10,8) obtained with ADAPT-VQE (solid blue) and Pruned-ADAPT-VQE (dashed orange) ansätze with qubit-excitation-based pool and prefactor = 0.05 for the N_2 molecule with interatomic distance of 2.0 Å, and computed with the 3-21G basis set and an active space of 8 orbitals with 2 frozen orbitals.

Figure S33 shows an example of a system where the pruning strategy leads to longer ansätze than the ADAPT-VQE, suggesting that for some exceptional cases, the hyperparameters should be further adjusted.

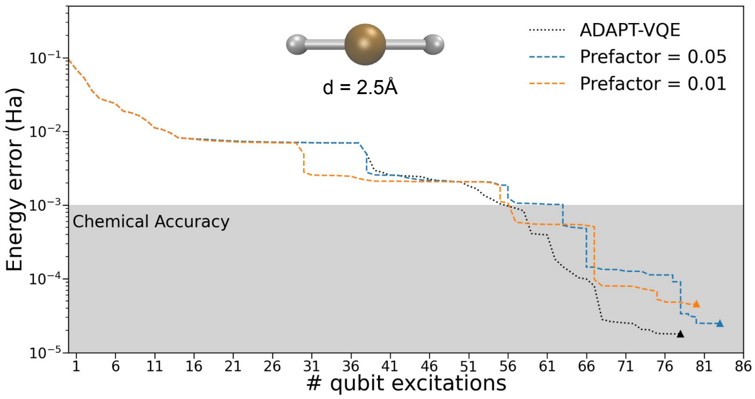

Figure S33: Energy errors (in Hartree) with respect to CASCI(4,8) obtained with ADAPT-VQE (dotted black) and Pruned-ADAPT-VQE (dashed blue for prefactor 0.05 and dashed orange for prefactor 0.01, see Section 5) ansätze for the BeH_2 molecule with interatomic distance of 2.5 Å, and computed with the 3-21G basis set and an active space of 8 orbitals plus one frozen orbital.

S10 STO-3G basis testing

This section includes simulations performed in STO-3G basis for both pools, fermionic and qubit-excitation-based.

S10.1 Fermionic pool

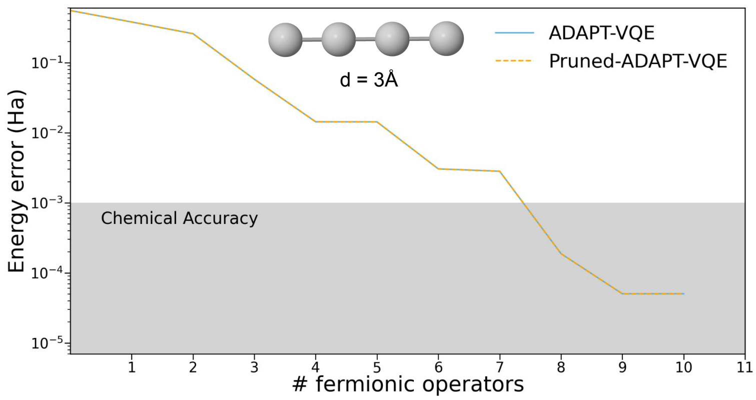

Figure S34: Energy errors (in Hartree) with respect to FCI obtained with ADAPT-VQE (solid blue) and Pruned-ADAPT-VQE (dashed orange) ansätze with fermionic pool for the linear H_4 molecule with interatomic distance of 3.0 Å, and computed with the STO-3G basis set and an active space of 8 orbitals.

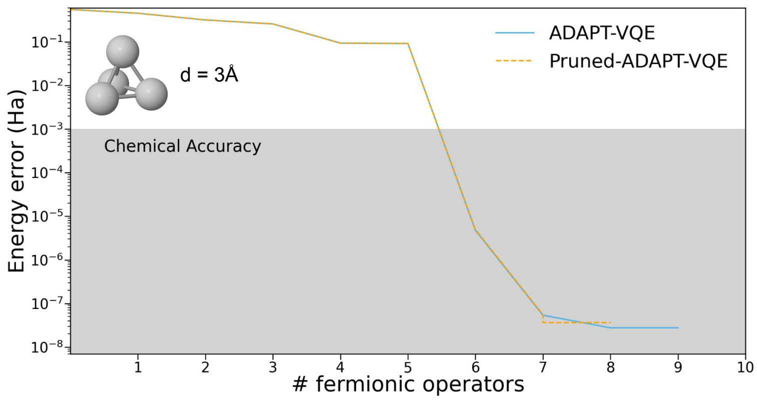

Figure S35: Energy errors (in Hartree) with respect to FCI obtained with ADAPT-VQE (solid blue) and Pruned-ADAPT-VQE (dashed orange) ansätze with fermionic pool for the tetrahedral H_4 molecule with interatomic distance of 3.0 Å, and computed with the STO-3G basis set and an active space of 8 orbitals.

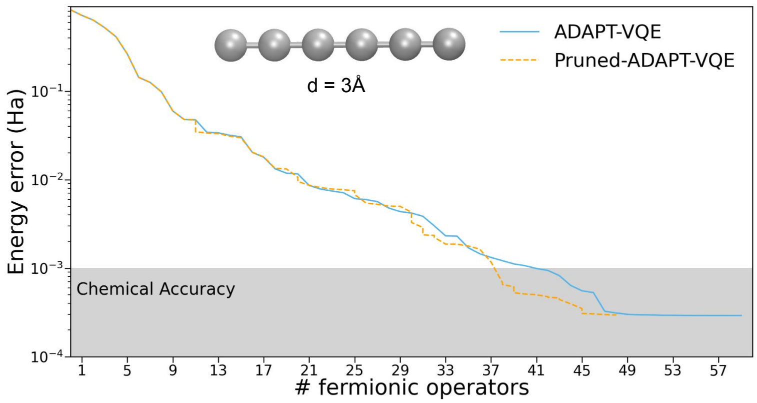

Figure S36: Energy errors (in Hartree) with respect to FCI obtained with ADAPT-VQE (solid blue) and Pruned-ADAPT-VQE (dashed orange) ansätze with fermionic pool for the H_6 molecule with interatomic distance of 3.0 Å, and computed with the STO-3G basis set and an active space of 6 orbitals.

S10.2 Qubit-excitation-based pool

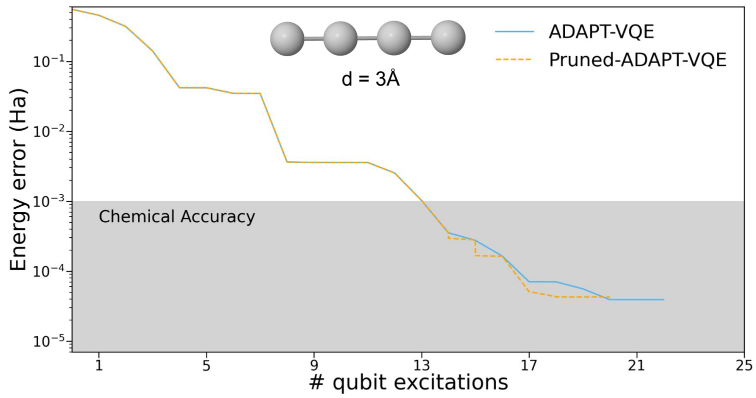

Figure S37: Energy errors (in Hartree) with respect to FCI obtained with ADAPT-VQE (solid blue) and Pruned-ADAPT-VQE (dashed orange) ansätze with qubit-excitation-based pool for the linear H_4 molecule with interatomic distance of 3.0 Å, and computed with the STO-3G basis set and an active space of 8 orbitals.

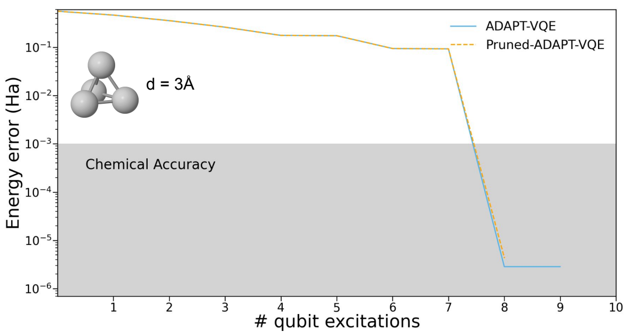

Figure S38: Energy errors (in Hartree) with respect to FCI obtained with ADAPT-VQE (solid blue) and Pruned-ADAPT-VQE (dashed orange) ansätze with qubit-excitation-based pool for the tetrahedral H_4 molecule with interatomic distance of 3.0 Å, and computed with the STO-3G basis set and an active space of 8 orbitals.

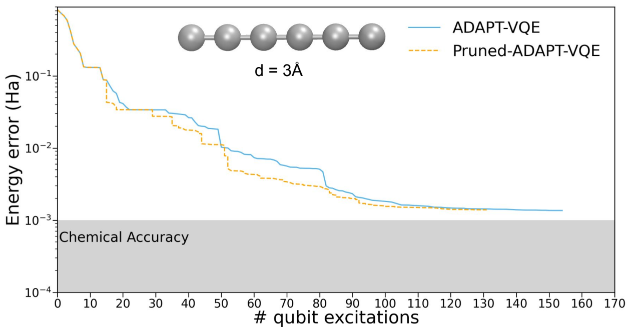

Figure S39: Energy errors (in Hartree) with respect to FCI obtained with ADAPT-VQE (solid blue) and Pruned-ADAPT-VQE (dashed orange) ansätze with qubit-excitation-based pool for the H_6 molecule with interatomic distance of 3.0 Å, and computed with the STO-3G basis set and an active space of 6 orbitals.

S11 Energy difference vs parameter value

In this section we show the relationship between the parameter values and its impact on the VQE energy for the linear H_4 molecule.

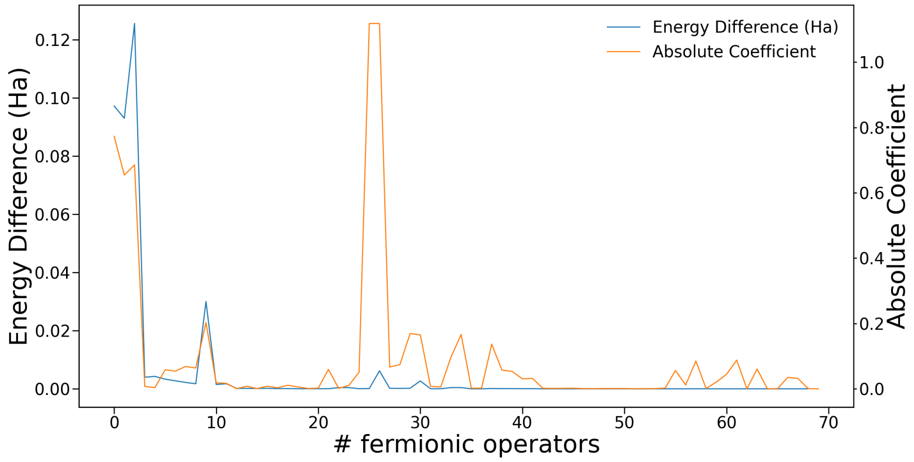

Figure S40: Energy differences (in a.u.) and parameter value for each added fermionic operator of ADAPT-VQE for the simulation of linear H_4 with the 3-21G basis set.

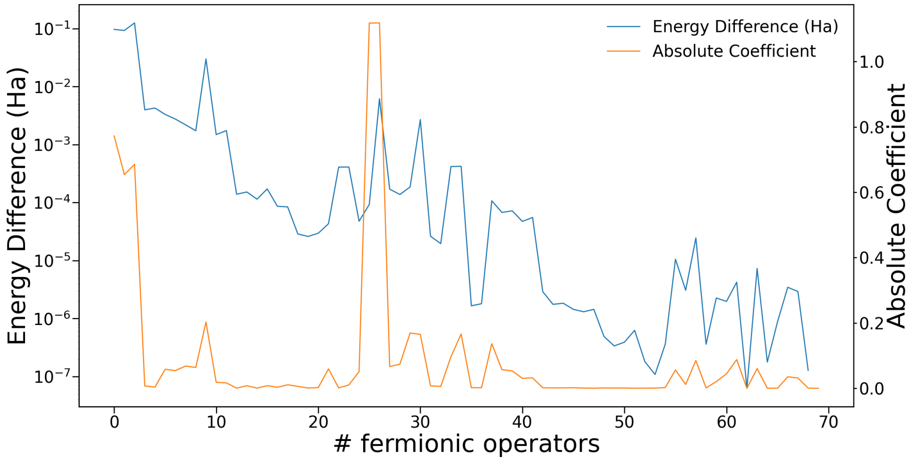

Figure S41: Energy differences (in a.u., logrithmic scale) and parameter value for each added fermionic operator of ADAPT-VQE for the simulation of linear H_4 with the 3-21G basis set.

S12 Gate counts

In this section, we compare Pruned-ADAPT-VQE and standard ADAPT-VQE by analyzing the number of CNOT gates in the ansatz at each iteration. The evaluation of the number of gates is performed by first mapping the fermionic operators to qubit operators using

the Jordan–Wigner transformation. The resulting qubit operators are then encoded into quantum circuits using the staircase algorithm. Circuit optimization is subsequently applied using the transpilation routines available in Qiskit to reduce the final CNOT count. The analyzed examples include the linear H_4 molecule using both the UCCSD ansatz and the qubit-excitation-based operator pool.

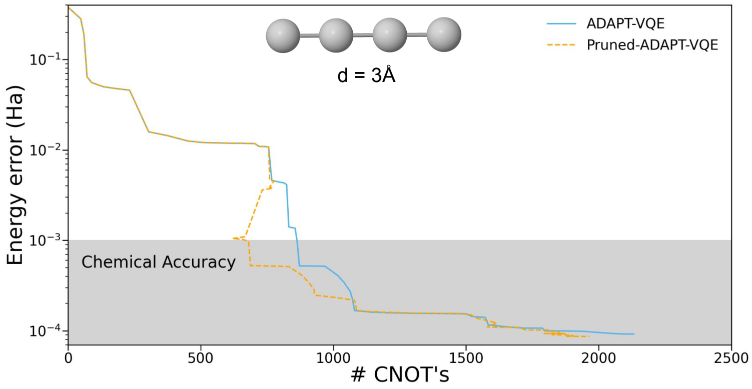

Figure S42: Energy errors (in Hartree) with respect to FCI as a function of the number of CNOT gates, obtained with ADAPT-VQE (solid blue) and Pruned-ADAPT-VQE (dashed orange) ansätze for the linear H_4 molecule with interatomic distance of 3.0 Å.

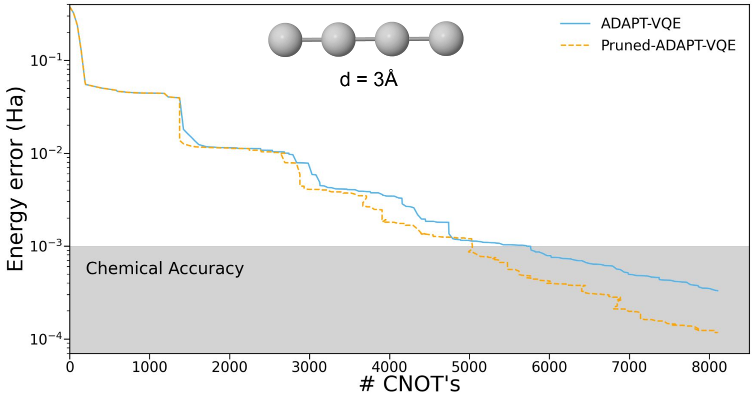

Figure S43: Energy errors (in Hartree) with respect to FCI as a function of the number of CNOT gates, obtained with ADAPT-VQE (solid blue) and Pruned-ADAPT-VQE (dashed orange) ansätze with the qubit-excitation-based pool operator for the linear H_4 molecule with interatomic distance of 3.0 Å.

S13 Impact of Removal on Classical Optimization Cost

In this section, we study whether the removal of an operator affects the next classical optimization loop, analyzing whether it leads to additional iterations.
The fairest comparison in this context is obtained by examining the optimization of the ansatz when the first operator is removed, since at that stage both ansätze are still identical. For the main simulation used as example in this work, H_4 with the fermionic pool, the first removal occurs at iteration 27, where Pruned-ADAPT-VQE deletes the operator in position 4. Afterward, both methods select the same operator at iteration 27 and again at iteration 28. Thus, by iteration 28 both ansätze are structurally the same, except that Pruned-ADAPT-VQE lacks the operator at position 4. In this iteration, ADAPT-VQE converges in 13 classical steps, whereas Pruned-ADAPT-VQE requires 14.

To give a broader view of the cost associated with operator removal, Figure S44 shows the energy error as a function of the cumulative number of energy evaluations performed by the classical optimizer throughout the algorithm. This simulation corresponds to the H_4 system at an interatomic distance of 3.0 Å with a fermionic operator pool. The figure shows that Pruned-ADAPT-VQE introduces a small overhead in some segments, and the two methods converge to similar results by the end of the simulation.

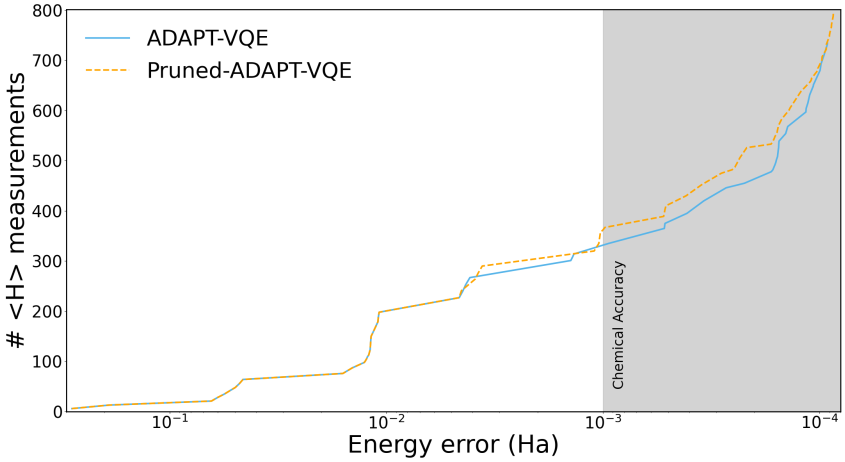

Figure S44: Number of energy measurements (or classical optimizer iterations) versus energy error (in Hartree) with respect to FCI for ADAPT-VQE (solid blue) and Pruned-ADAPT-VQE (dashed orange) ansätze with the fermionic pool for the linear H_4 molecule with interatomic distance of 3.0 Å.

References

(S1) Yordanov, Y. S.; Armaos, V.; Barnes, C. H. W.; Arvidsson-Shukur, D. R. M. Qubit-excitation-based adaptive variational quantum eigensolver. *Commun. Phys.* **2021**, *4*, 228.
